# Supplementary material for: Silver Diamine Fluoride (SDF) Efficacy in Arresting Cavitated Caries Lesions in Primary Molars: A Systematic Review and Metanalysis
Source: Int J Environ Res Public Health. 2022 Oct 9;19(19):12917. doi: 10.3390/ijerph191912917 (PMC9566773; doi:10.3390/ijerph191912917)
Supplement: Supplementary file 1 [file ijerph-19-12917-s001.zip › ijerph-1916560-supplementary.pdf]

Table S1. Extraction form ………………………………………………………………………………………. 1

Table S2. Studies excluded after title and abstract screening ……………………………… 4

Table S3. Studies studies excluded after full-text reading …………………………………..48

Table S4. Summary statistics, results for each study (fixed-effect model) …………. 50

Table S1. Extraction form.

	Publication Year					
	Author					
	Title					
	Item Type (Article, Thesis, Congress presentation..)					
	Journal					
	Type of study (RCT, retrospettivo,…)					
N° of patient treated	Total number of patient					
	Treated					

	Control					
N° of POSTERIOR tooth treated	Total number of POSTERIOR tooth					
	Treated					
	Control					
**Baseline characteristics** (if data available, divided based on groups)	Patient's age range					
	n° female and male					
	ICDAS/dmft					
Study Protocol	Primary outcome					
	Secondary outcome					
	TEST GROUP (SDF+Evtl other treatment)					
	TEST GROUP Follow up (mo./yy)					

	CONTROL GROUP (no treatment/ ART/ fluoride varnish)					
	CONTROL GROUP Follow up (mo./yy)					
	Setting (dental clinic, school..)					
	Operator (dentist, hygienist, ….)					
	Blinding					
	Esame					
Results	TEST GROUP					
	TEST GROUP p-value					
	CONTROL GROUP					
	CONTROL GROUP p-value					
	Country					

	Database					

Table S2. Studies excluded after title and abstract screening.

Publication Year	Author Names	Title	Source title	Reason for exclusion
2016	Duangthip D, Jiang M, Chu CH, Lo EC.	Restorative approaches to treat dentin caries in preschool children: systematic review	European Journal of Paediatric dentistry	Different type of study
2020	Splieth CH, Banerjee A, Bottenberg P, Breschi L, Campus G, Ekstrand KR, Giacaman RA, Haak R, Hannig M, Hickel R, Juric H, Lussi A, Machiulskiene V, Manton DJ, Jablonski-Momeni A, Opdam NJM, Paris S, Santamaría RM, Schwendicke F, Tassery H, Ferreira Zandona A, Zero DT, Zimmer S, Doméjean S.	How to Intervene in the Caries Process in Children: A Joint ORCA and EFCD Expert Delphi Consensus Statement	Caries research	Different type of study
2020	Santamaría RM, Abudrya MH, Gül G, Mourad MS, Gomez GF, Zandona AGF.	How to Intervene in the Caries Process: Dentin Caries in Primary Teeth	Caries research	Different type of study
2021	Al Masri A, Abudrya MEH, Splieth CH, Schmoeckel J, Mourad MS, Santamaría RM.	How did the COVID-19 pandemic lockdown affect dental emergency care in children? Retrospective study in a specialized pedodontic center	Quintessence Int	Different outcome
2017	[No author name available]	JUCSF protocol for caries arrest using silver diamine fluoride: rationale, indications, and consent	British dental journal	Different type of study
2020	Aarthi S., Arangannal P., Jeevarathan J., Vijayakumar M., Aarthi J., Amudha S.	The decision maker - An insight on factors influencing parental acceptance of utilising sdf treatment for their children	European Journal of Molecular and Clinical Medicine	Different outcome

2021	Abdelaziz M, Yang V, Chang N, Darling C, Fried W, Seto J, Fried D.	Monitoring silver diamine fluoride application with optical coherence tomography	Proc SPIE Int Soc Opt Eng	Different type of study
2020	Abdullah N, Al Marzooq F, Mohamad S, Abd Rahman N, Rani KGA, Chi Ngo H, Samaranayake LP.	The antibacterial efficacy of silver diamine fluoride (SDF) is not modulated by potassium iodide (KI) supplements: A study on in-situ plaque biofilms using viability real-time PCR with propidium monoazide	PLoS One	Different type of sample
2011	Aboushelib M.N.	Clinical performance of self-etching adhesives with saliva contamination.	The journal of adhesive dentistry	Serch noise
1996	Afonso F, Gotjamanos T.	An in vitro study of the distribution of silver and fluoride following application of 40 per cent silver fluoride solution to dentine	Aust Dent J	Different type of study
2021	Ahmed AM, Abdellatif HM, Baghdady SI, Abdelaziz WE, Elkateb MA.	Child Discomfort and Parental Acceptability of Silver Diamine Fluoride and Alternative Restorative Treatment: A Randomized Controlled Clinical Trial	J Dent	Different outcome
2019	Ahmed, F., Prashanth, S., Sindhu, K., Nayak, A., Chaturvedi, S.	Antimicrobial efficacy of nanosilver and chitosan against Streptococcus mutans, as an ingredient of toothpaste formulation: An in vitro study	Journal of Indian Society of Pedodontics and Preventive Dentistry	Serch noise
2020	Ahsana A., Gurunathan D.	Effect of silver diamine fluoride treatment on the quality of life of children	Indian Journal of Forensic Medicine and Toxicology	Different outcome
2019	Akyildiz M, SÃ¶nmez IS.	Comparison of Remineralising Potential of Nano Silver Fluoride, Silver Diamine Fluoride and Sodium Fluoride Varnish on Artificial Caries: An In Vitro Study	Oral Health Prev Dent	Different type of study
2021	Al Habdan, A.H., Al Awdah, A., Aldosari, G., Almogbel, S., Alawaji, R.	Caries arrest using silver diamine fluoride: Knowledge, attitude, and perception of adult patients in Saudi Arabia	Saudi Dental Journal	Different outcome
2020	Alajlan, G., Alshaikh, H., Alshamrani, L., Alanezi, M., Alarfaj, S., Alswayyed, T.	Knowledge on and attitude toward silver diamine fluoride among Saudi dental practitioners in Riyadh public hospitals	Clinical, Cosmetic and Investigational Dentistry	Different outcome
2019	Al-Angari SS, Lippert F, Platt JA, Eckert GJ, GonzÃ¡lez-Cabezas C, Li Y, Hara AT.	Bleaching of simulated stained-remineralized caries lesions in vitro	Clin Oral Investig	Different type of study

2021	Aldhaian, B.A., Balhaddad, A.A., Alfaifi, A.A., Levon, J.A., Eckert, G.J., Hara, A.T., Lippert, F.	In vitro demineralization prevention by fluoride and silver nanoparticles when applied to sound enamel and enamel caries-like lesions of varying severities	Journal of Dentistry	Different type of study
2021	Aldhuwayhi, S.	Silver diamine fluoride in reducing dentin hypersensitivity in vital tooth preparation: A case report	International Journal of Dentistry and Oral Science	Different outcome
2021	Ali, A., Ismail, H., Amin, K.	Effect of nanosilver mouthwash on prevention of white spot lesions in patients undergoing fixed orthodontic treatment - a randomized double-blind clinical trial	Journal of Dental Sciences	Serch noise
2019	Allen PF, Da Mata C, Hayes M.	Minimal intervention dentistry for partially dentate older adults	Gerodontology	Different type of sample
2021	Almarwan M, Almawash A, AlBrekan A, Albluwi S.	Parental Acceptance for the Use of Silver Diamine Fluoride on Their Special Health Care-Needs Child's Primary and Permanent Teeth	Clin Cosmet Investig Dent	Different outcome
2020	Al-Nerabieah, Z., Arrag, E.A., Rajab, A.	Cariostatic efficacy and children acceptance of nano-silver fluoride versus silver diamine fluoride: A randomized controlled clinical trial	Open Journal of Stomatology	Follow up < 12 mo.
2020	Alshahni R.Z., Alshahni M.M., Hiraishi N., Makimura K., Tagami J.	Effect of Silver Diamine Fluoride on Reducing Candida albicans Adhesion on Dentine	Mycopathologia	Different outcome
2019	Alshammari AF, Almuqrin AA, Aldakhil AM, Alshammari BH, Lopez JNJ.	Parental perceptions and acceptance of silver diamine fluoride treatment in Kingdom of Saudi Arabia	Int J Health Sci (Qassim)	Different outcome
2013	Alves, F.B.T., Lenzi, T.L., Reis, A., Loguercio, A.D., Carvalho, T.S., Raggio, D.P.	Bonding of simplified adhesive systems to caries-affected dentin of primary teeth	Journal of Adhesive Dentistry	Serch noise
2010	Alves, T.M.S., Silva, C.A., da Silva, N.B., de Medeiros, E.B., ValenÃ§a, A.M.G.	Antimicrobial activity of fluoridated products on biofilm-forming bacteria: An in vitro study	Pesquisa Brasileira em Odontopediatria e Clinica Integrada	Different outcome
2019	Antonioni MB, Fontana M, Salzmann LB, Inglehart MR.	Pediatric Dentists' Silver Diamine Fluoride Education, Knowledge, Attitudes, and Professional Behavior: A National Survey	J Dent Educ	Different type of study

2006	Ariffin, Z., Ngo, H., McIntyre, J.	Enhancement of fluoride release from glass ionomer cement following a coating of silver fluoride	Australian Dental Journal	Different type of study
2021	Arnaud M, Junior PC, Lima MG, E Silva AV, Araujo JT, Gallembeck A, de FranÃ§a Caldas JÃºnior A, Rosenblatt A.	Nano-silver Fluoride at Higher Concentration for Caries Arrest in Primary Molars: A Randomized Controlled Trial	Int J Clin Pediatr Dent	Serch noise
1995	Aron V.O.	Porcelain veneers for primary incisors: a case report.	Quintessence international (Berlin, Germany : 1985)	Serch noise
2012	Ashkenazi, M., Bidoosi, M., Levin, L.	Factors associated with reduced compliance of children to dental preventive measures	Odontology	Serch noise
2020	Asif A., Gurunathan D.	Parental acceptance of silver diamine fluoride treatment for children	International Journal of Research in Pharmaceutical Sciences	Different outcome
2008	Awliya, W.Y., El-Sahn, A.M.	Leakage pathway of Class V cavities restored with different flowable resin composite restorations	Operative Dentistry	Serch noise
2019	Bagher S.M., Sabbagh H.J., Aljohani S.M., Alharbi G., Aldajani M., Elkhodary H.	Parental acceptance of the utilization of silver diamine fluoride on their child's primary and permanent teeth	Patient Preference and Adherence	Different type of study
2021	Bahathiq, A., Arafa, A.	Factors modulating parental acceptance of SDF treatment	Pediatric Dental Journal	Different outcome
2019	Bakhsh, T.A., Altouki, N.H., Baeesa, L.S., Baamer, R.A., Alshebany, R.M., Natto, Z., Nasir, A., Turkistani, A., Hasanain, F., Naguib, G.H.	Effect of self-etch adhesives on the internal adaptation of composite restoration: a CP-OCT Study	Odontology	Serch noise
2015	Barrak, I., UrbÃ¡n, E., TurzÃ³, K., Nagy, K., Braunitzer, G., StÃ¡jer, A.	Short- and long-term influence of fluoride-containing prophylactics on the growth of streptococcus mutans on titanium surface	Implant Dentistry	Serch noise
1984	Barreiro Davina A, Alvarez Brasa C.	Dentin remineralization	Rev Actual Estomatol Esp	Abstract or full-text not avable

2017	Barreto, K.A., dos Prazeres, L.D.K.T., Lima, D.S.M., Redivivo, R.M.M.P., Colares, V.	Children's anxiety during dental treatment with minimally invasive approaches: Findings of an analytical cross-sectional study	Pesquisa Brasileira em Odontopediatria e Clinica Integrada	Different outcome
2010	Beltra¡n-Aguilar ED.	Silver diamine fluoride (SDF) may be better than fluoride varnish and no treatment in arresting and preventing cavitated carious lesions	J Evid Based Dent Pract	Different type of study
1988	Berg, J.H., Donly, K.J., Posnick, W.R.	Glass ionomer-silver restorations: a demineralization-remineralization concept.	Quintessence international	Serch noise
1990	Berg, J.H., Farrell, J.E., Brown, L.R.	Class II glass ionomer/silver cermet restorations and their effect on interproximal growth of mutans streptococci.	Pediatric dentistry	Serch noise
2020	Bernabe E, Marcenes W.	Can minimal intervention dentistry help in tackling the global burden of untreated dental caries?	Br Dent J	Different type of study
1986	Billings, R.J.	Restoration of Carious Lesions of the Root	Gerodontology	Serch noise
2018	Bimstein E., Damm D.	Human Primary Tooth Histology Six Months after Treatment with Silver Diamine Fluoride	The Journal of clinical pediatric dentistry	Different outcome
2009	Bonifacio, C.C., Navarro, R.S., Sardenberg, F., Imparato, J.C.P., de Carvalho, R.C.R., Raggio, D.P.	Microleakage of an adhesive system used as a fissure sealant	Journal of Contemporary Dental Practice	Serch noise
2016	Bowen DM.	Effectiveness of Professionally-Applied Silver Diamine Fluoride in Arresting Dental Caries	J Dent Hyg	Different type of study
2009	Braga MM, Mendes FM, De Benedetto MS, Imparato JC.	Effect of silver diammine fluoride on incipient caries lesions in erupting permanent first molars: a pilot study	J Dent Child (Chic)	Different outcome
2020	Braz, P.V.F., Dos Santos, A.F.L., Leal, S.C., Pereira, P.N.R., Ribeiro, A.P.D.	The effect of silver diamine fluoride and cleaning methods on bond strength of glass-ionomer cements to caries-affected dentin	American Journal of Dentistry	Different type of study

2020	Braz, P.V.F., Dos Santos, A.F.L., Pereira, P.N.R., Ribeiro, A.P.D.	Silver diamine fluoride and cleaning methods effects on dentin bond strength	American Journal of Dentistry	Different outcome
2021	Bridge, G., Martel, A.-S., Lomazzi, M.	Silver Diamine Fluoride: Transforming Community Dental Caries Program: THE USE OF SDF IN COMMUNITY CARIES PROGRAMMES	International Dental Journal	Different type of study
2019	Brignardello-Petersen R.	Silver diamine fluoride seems to be effective in preventing and arresting root caries in older adults compared with placebo, but there is very low certainty in the magnitude of the benefit	J Am Dent Assoc	Different outcome
2017	Brignardello-Petersen R.	37% silver diamine fluoride is more effective than 12% silver diamine fluoride in arresting caries in the primary dentition	J Am Dent Assoc	Different type of study
2018	Brignardello-Petersen R.	Although silver diamine fluoride arrested caries sooner than fluoride varnish, the overall proportion of arrested caries was not importantly different after 30 months	J Am Dent Assoc	Different type of study
2018	Brignardello-Petersen R.	Increasing concentration and application frequency of silver diamine fluoride results in more arrested carious lesions being stained black but no other important adverse effects	J Am Dent Assoc	Different type of study
2021	Brondani MA, Siarkowski M, Alibrahim I, Ribeiro CCC, Alves CMC, Donnelly LR, Mathu-Muju K.	An Overview of Pedagogical Approaches to Caries-Control Medications in Canadian Dental and Dental Hygiene Programs	J Can Dent Assoc	Serch noise
2018	Burgess JO, Vaghela PM.	Silver Diamine Fluoride: A Successful Anticarious Solution with Limits	Adv Dent Res	Different type of study
2019	Burgette J.M., Weintraub J.A., Birken S.A., Lewis T.A., White B.A.	Development of a Silver Diamine Fluoride Protocol in Safety Net Dental Settings	Journal of dentistry for children (Chicago, Ill.)	Different outcome
2015	Burns J, Hollands K.	Nano Silver Fluoride for preventing caries	Evid Based Dent	Serch noise
2020	C C BO, A R VO, R Y SB, M A AT.	Study of Demineralized Dental Enamel Treated with Different Fluorinated Compounds by Raman Spectroscopy	J Biomed Phys Eng	Different type of study

2019	Cai J, Burrow MF, Manton DJ, Tsuda Y, Sobh EG, Palamara JEA.	Effects of silver diamine fluoride/potassium iodide on artificial root caries lesions with adjunctive application of proanthocyanidin	Acta Biomater	Serch noise
2015	Calarco, A., Di Salle, A., Tammaro, L., De Luca, I., Mucerino, S., Petillo, O., Riccitiello, F., Vittoria, V., Peluso, G.	Long-term fluoride release from dental resins affects STRO-1+ cell behavior	Journal of Dental Research	Serch noise
2018	Canares G, Hsu KL, Dhar V, Katechia B.	Evidence-based care pathways for management of early childhood caries	Gen Dent	Different type of study
1971	Canton A.	Topical application of a silver nitrate ammonia solution of high concentration	Mondo odontostomatologico	Abstract or full-text not avable
2015	Carrera, C.A., Lan, C., Escobar-Sanabria, D., Li, Y., Rudney, J., Aparicio, C., Fok, A.	The use of micro-CT with image segmentation to quantify leakage in dental restorations	Dental Materials	Serch noise
2021	Castelo R, Attik N, Catirse ABCEB, Pradelle-Plasse N, Tirapelli C, Grosgogeat B.	Is there a preferable management for root caries in middle-aged and older adults? A systematic review	Br Dent J	Different outcome
2019	Cernigliaro D., Kumar A., Northridge M.E., Wu Y., Troxel A.B., Cunha-Cruz J., Balzer J., Okuji D.M.	Caregiver satisfaction with interim silver diamine fluoride applications for their children with caries prior to operating room treatment or sedation	Journal of public health dentistry	Different outcome
2015	Chaple Gil, A.M.	Effect of the main materials used in dental practice against the formation of bacterial dental plaque	Revista Cubana de Estomatologia	Serch noise
2018	Chen K.J., Gao S.S., Duangthip D., Lo E.C.M., Chu C.H.	The caries-arresting effect of incorporating functionalized tricalcium phosphate into fluoride varnish applied following application of silver nitrate solution in preschool children: Study protocol for a randomized, double-blind clinical trial	Trials	Different type of study
2021	Chen K.J., Gao S.S., Duangthip D., Lo E.C.M., Chu C.H.	Randomized Clinical Trial on Sodium Fluoride with Tricalcium Phosphate	Journal of Dental Research	Serch noise
2020	Chen KF, Milgrom P, Lin YS.	Silver Diamine Fluoride in Children Using Physiologically Based PK Modeling	J Dent Res	Different outcome

2018	Chen KJ, Gao SS, Duangthip D, Lo ECM, Chu CH.	Managing Early Childhood Caries for Young Children in China	Healthcare (Basel)	Different type of study
2012	Cheng, L., Weir, M.D., Xu, H.H.K., Antonucci, J.M., Kraigsley, A.M., Lin, N.J., Lin-Gibson, S., Zhou, X.	Antibacterial amorphous calcium phosphate nanocomposites with a quaternary ammonium dimethacrylate and silver nanoparticles	Dental Materials	Serch noise
2017	Chhokar SK, Laughter L, Rowe DJ.	Perceptions of Registered Dental Hygienists in Alternative Practice Regarding Silver Diamine Fluoride	J Dent Hyg	Different type of study
2018	Chi DL, Milgrom P, Gillette J.	Engaging Stakeholders in Patient-Centered Outcomes Research Regarding School-Based Sealant Programs	J Dent Hyg	Serch noise
2011	Chibinski A.C., Stanislawczuk R., Roderjan D.A., Loguercio A.D., Wambier D.S., Grande R.H.M., Reis A.	Clinical versus laboratory adhesive performance to wet and dry demineralized primary dentin	American Journal of Dentistry	Serch noise
2017	Chibinski A.C., Wambier L.M., Feltrin J., Loguercio A.D., Wambier D.S., Reis A.	Silver Diamine Fluoride Has Efficacy in Controlling Caries Progression in Primary Teeth: A Systematic Review and Meta-Analysis	Caries research	Different type of study
2015	Chu C.-H., Gao S.S., Li S.K.Y., Wong M.C.M., Lo E.C.M.	The effectiveness of the biannual application of silver nitrate solution followed by sodium fluoride varnish in arresting early childhood caries in preschool children: Study protocol for a randomised controlled trial	Trials	Different type of study
2008	Chu C.H., Lo E.C.M.	Microhardness of dentine in primary teeth after topical fluoride applications	Journal of Dentistry	Different outcome
2002	Chu C.H., Lo E.C.M., Lin H.C.	Effectiveness of silver diamine fluoride and sodium fluoride varnish in arresting dentin caries in Chinese pre-school children	Journal of Dental Research	Different outcome
2014	Chu CH, Lee AH, Zheng L, Mei ML, Chan GC.	Arresting rampant dental caries with silver diamine fluoride in a young teenager suffering from chronic oral graft versus host disease post-bone marrow transplantation: a case report	BMC Res Notes	Different type of sample
2008	Chu CH, Lo EC.	Promoting caries arrest in children with silver diamine fluoride: a review	Oral Health Prev Dent	Different type of study

2012	Chu CH, Mei L, Seneviratne CJ, Lo EC.	Effects of silver diamine fluoride on dentine carious lesions induced by Streptococcus mutans and Actinomyces naeslundii biofilms	Int J Paediatr Dent	Different type of study
2003	Civelek, A., Ersoy, M., L'Hotelier, E., Soymano, M., Say, E.C.	Polymerization shrinkage and microleakage in Class II cavities of various resin composites	Operative Dentistry	Serch noise
2020	Colombini-Ishikiriama BL, Dionisio TJ, Garbieri TF, da Silva RA, Machado MAAM, de Oliveira SHP, Lara VS, Greene AS, Santos CF.	What is the response profile of deciduous pulp fibroblasts stimulated with E. coli LPS and E. faecalis LTA?	BMC Immunol	Different type of study
2017	Contreras V, Toro MJ, Elias-Boneta AR, Encarnacion-Burgos A.	Effectiveness of silver diamine fluoride in caries prevention and arrest: a systematic literature review	Gen Dent	Different type of study
2020	Correa-Faria, P., Viana, K.A., Raggio, D.P., Hosey, M.T., Costa, L.R.	Recommended procedures for the management of early childhood caries lesions-A scoping review by the Children Experiencing Dental Anxiety: Collaboration on Research and Education (CEDACORE)	BMC Oral Health	Different type of study
2011	Costa JF, Siqueira WL, Loguercio AD, Reis A, Oliveira Ed, Alves CM, Bauer JR, Grande RH.	Characterization of aqueous silver nitrate solutions for leakage tests	J Appl Oral Sci	Serch noise
2013	Craig G.G., Powell K.R., Price C.A.	Clinical evaluation of a modified silver fluoride application technique designed to facilitate lesion assessment in outreach programs.	BMC oral health	Serch noise
1981	Craig, G.G., Powell, K.R., Cooper, M.H.	Caries progression in primary molars: 24-month results from a minimal treatment programme	Community Dentistry and Oral Epidemiology	Different outcome
2018	Crete P, Boyd LD, Fitzgerald JK, LaSpina LM.	Access to Preventive Oral Health Services for Homebound Populations: A pilot program	J Dent Hyg	Serch noise
2021	Crisp J., Mihas P., Sanders A.E., Divaris K., Wright J.T.	Influences on dentists' adoption of nonsurgical caries management techniques: A qualitative study	Journal of the American Dental Association (1939)	Different type of study
2020	Croll TP, Berg J.	Delivery Methods of Silver Diammine Fluoride to Contacting Proximal Tooth Surfaces and History of Silver in Dentistry	Compend Contin Educ Dent	Different type of study

2021	Crowder L.	Management options of early childhood caries	Evid Based Dent	Different type of study
2020	Crystal Y.O., Janal M.N., Yim S., Nelson T.	Teaching and utilization of silver diamine fluoride and Hall-style crowns in US pediatric dentistry residency programs	Journal of the American Dental Association (1939)	Different outcome
2019	Crystal Y.O., Rabieh S., Janal M.N., Rasamimari S., Bromage T.G.	Silver and fluoride content and short-term stability of 38% silver diamine fluoride	Journal of the American Dental Association (1939)	Different outcome
2018	Crystal YO, Chaffee BW.	Silver Diamine Fluoride is Effective in Arresting Caries Lesions in Primary Teeth	J Evid Based Dent Pract	Different type of study
2017	Crystal YO, Janal MN, Hamilton DS, Niederman R.	Parental perceptions and acceptance of silver diamine fluoride staining	J Am Dent Assoc	Different outcome
2019	Crystal YO, Niederman R.	Evidence-Based Dentistry Update on Silver Diamine Fluoride	Dent Clin North Am	Different type of study
2016	Crystal YO, Niederman R.	Silver Diamine Fluoride Treatment Considerations in Children's Caries Management	Pediatr Dent	Different type of study
2019	Crystal, Y.O.	Silver diamine fluoride (SDF): Its role in caries management	Dental Update	Different type of study
2019	Crystal, Y.O., Kreider, B., Raveis, V.H.	Parental expressed concerns about silver diamine fluoride (SDF) treatment	Journal of Clinical Pediatric Dentistry	Different outcome
2021	Cunha-Cruz J, Pires Dos Santos AP.	Professionally and Self-Applied Fluorides are Effective in Preventing Dental Root Caries	J Evid Based Dent Pract	Serch noise
2021	da Cunha WA, Palma LF, Shitsuka C, CorrÃªa FNP, Duarte DA, CorrÃªa MSNP.	Efficacy of silver diamine fluoride and sodium fluoride in inhibiting enamel erosion: an ex vivo study with primary teeth	Eur Arch Paediatr Dent	Different type of study

2020	Dang C, Comnick CL, Tabrizi M, Kaufman L, Soto A, Smith BM, Ribeiro APD, Tobey T, Capin OR, Scully A, Prince D, Xie XJ, Marchini L.	Assessment of knowledge and perception about silver diamine fluoride (SDF) for treating older adults among graduating dental students	J Dent Educ	Different type of sample
2020	Davis M.R., Johnson E.L., Meyer B.D.	Comparing Dental Treatment between Children Receiving and not Receiving Silver Diamine Fluoride	The Journal of clinical pediatric dentistry	Different outcome
2014	De Alencar, N.A., Fidalgo, T.K.S., Cajazeira, M.R.R., Maia, L.C.	Influence of the number of adhesive layers on adhesive interface properties under cariogenic challenge using streptococcus mutans	Journal of Adhesive Dentistry	Serch noise
2020	de Siqueira, F.S.F., Morales, L.A.R., Granja, M.C.P., de Oliveira de Melo, B., Monteiro-Neto, V., Reis, A., Cardenas, A.F.M., Loguercio, A.D.	Effect of silver diamine fluoride on the bonding properties to caries-affected dentin	Journal of Adhesive Dentistry	Different outcome
2021	de Souza BM, Silva MS, Braga AS, Bueno PSK, da Silva Santos PS, Buzalaf MAR, MagalhÃ£es AC.	Protective effect of titanium tetrafluoride and silver diamine fluoride on radiation-induced dentin caries in vitro	Sci Rep	Different outcome
2009	Deery, C.	Silver lining for caries cloud?	Evidence-Based Dentistry	Different type of study
2006	Delbem, A.C.B., Bergamaschi, M., Sassaki, K.T., Cunha, R.F.	Effect of fluoridated varnish and silver diamine fluoride solution on enamel demineralization: pH-cycling study	Journal of Applied Oral Science	Different type of study
2021	Detsomboonrat, P., Thongmak, P., Lertpayab, P., Aiemsri, W., Sooampon, S.	Optimal concentration of potassium iodide to reduce the black staining of silver diamine fluoride	Journal of Dental Sciences	Different outcome
2016	Deutsch A.	An alternate technique of care using silver fluoride followed by stannous fluoride in the management of root caries in aged care	Spec Care Dentist	Different type of sample
2018	Deutsch AA, Craig GG.	Unexpected sequel to silver fluoride followed by stannous fluoride treatment of root stumps supporting an overlay denture in an aged-care patient	Spec Care Dentist	Different outcome
2018	Devji T.	Silver diamine fluoride is probably more effective than atraumatic restorative treatment, fluoride varnish, or no treatment for controlling caries progression in children	J Am Dent Assoc	Different type of study

1998	Dionysopoulos P, Kotsanos N, Papadogiannis Y, Konstantinidis A.	Artificial caries formation around fluoride-releasing restorations in roots	J Oral Rehabil	Serch noise
1994	Dionysopoulos, P., Kotsanos, N., Koliniotou-Koubia, Papagodiannis, Y.	Secondary caries formation in vitro around fluoride-releasing restorations.	Operative dentistry	Serch noise
1991	Dodds, M.W., Edgar, W.M.	Interactions between fluoride and plaque in the remineralization of enamel caries-like lesions.	American journal of dentistry	Different outcome
1990	Donly, K.J., Wild, T.W., Jensen, M.E.	Posterior composite Class II restorations: in vitro comparison of preparation designs and restoration techniques	Dental Materials	Serch noise
2012	Dos Santos Jr., V.E., De Vasconcelos, F.M.N., De Souza, P.R., Ribeiro, A.G., Rosenblatt, A.	Adverse events on the use of interim therapeutic in schoolchildren: Silver diamine fluoride interim therapeutic restorative - A pilot study	Revista Odonto Ciencia	Different outcome
2017	Duangthip D, Chen KJ, Gao SS, Lo ECM, Chu CH.	Managing Early Childhood Caries with Atraumatic Restorative Treatment and Topical Silver and Fluoride Agents	Int J Environ Res Public Health	Different type of study
2015	Duangthip D, Jiang M, Chu CH, Lo EC.	Non-surgical treatment of dentin caries in preschool children--systematic review	BMC Oral Health	Different type of study
2018	Duangthip D., Fung M.H.T., Wong M.C.M., Chu C.H., Lo E.C.M.	Adverse Effects of Silver Diamine Fluoride Treatment among Preschool Children	Journal of dental research	Different outcome
2019	Duangthip D., Gao S.S., Chen K.J., Lo E.C.M., Chu C.H.	Oral health-related quality of life of preschool children receiving silver diamine fluoride therapy: A prospective 6-month study	Journal of dentistry	Different outcome
2016	Duangthip, D., Jiang, M., Chu, C.H., Lo, E.C.M.	Restorative approaches to treat dentin caries in preschool children: Systematic review	European Journal of Paediatric Dentistry	Different type of study
2007	Duarte Jr., S., Dinelli, W., Carmona Da Silva, M.H.	Influence of resin composite insertion technique in preparations with a high C-factor	Quintessence International	Serch noise

2008	Duarte Jr., S., Saad, J.R.C.	Marginal adaptation of Class 2 adhesive restorations	Quintessence International	Serch noise
2012	Duffin, S.	Back to the future: the medical management of caries introduction.	Journal of the California Dental Association	Different type of study
2021	Durrani, F., Painuly, H., Shukla, A., Nahid, R., Pandey, S.	Terminal dentition to functional esthetic full-mouth implant reconstruction: Evidence-based approach	Journal of Indian Society of Periodontology	Serch noise
2008	Eden E., Topaloglu-Ak V., Cuijpers V., Frencken J.E.	Micro-CT for measuring marginal leakage of Class II resin composite restorations in primary molars prepared in vivo	American Journal of Dentistry	Different type of study
2021	Erdwey D, Meyer-Lueckel H, Esteves-Oliveira M, Apel C, Wierichs RJ.	Demineralization Inhibitory Effects of Highly Concentrated Fluoride Dentifrice and Fluoride Gels/Solutions on Sound Dentin and Artificial Dentin Caries Lesions in vitro	Caries Res	Different type of study
2021	Ezzeldin, T., Al-Awasi, K.A., Bader, R.M., Alshaikhi, A.Y., Hakami, A.H., Siddiqui, I.A., Almulhim, A.A., Alsubaie, T.M.	A Study to assess the awareness and use of Silver Diammine Fluoride and Hall Technique among dental professionals and dental students in the Eastern Province	Saudi Dental Journal	Different type of study
2020	Fakhruddin KS, Egusa H, Ngo HC, Panduwawala C, Pesee S, Samaranayake LP.	Clinical efficacy and the antimicrobial potential of silver formulations in arresting dental caries: a systematic review	BMC Oral Health	Different type of study
2020	Fakhruddin KS, Egusa H, Ngo HC, Panduwawala C, Pesee S, Venkatachalam T, Samaranayake LP.	Silver diamine fluoride (SDF) used in childhood caries management has potent antifungal activity against oral Candida species	BMC Microbiol	Different outcome
2014	Faller, R.V., Eversole, S.L., Saunders-Burkhardt, K.	Protective benefits of a stabilised stannous-containing fluoride dentifrice against erosive acid damage.	International dental journal	Serch noise
2011	Ferreira, S.Q., Da Costa, T.R.F., Klein-Janior, C.A., De Lourdes Rodrigues Accorinte, M., Meier, M.M., Loguercio, A.D., Reis, A.	Improvement of exposure times: Effects on adhesive properties and resin-dentin bond strengths of etch-and-rinse adhesives	Journal of Adhesive Dentistry	Serch noise
2020	Firouzmandi M, Mohaghegh M, Jafarpisheh M.	Effect of silver diamine fluoride on the bond durability of normal and carious dentin	J Clin Exp Dent	Different type of study

2019	Firouzmandi, M., Shafiei, F., Jowkar, Z., Nazemi, F.	Effect of Silver Diamine Fluoride and Proanthocyanidin on Mechanical Properties of Caries-Affected Dentin	European Journal of Dentistry	Different type of study
1990	FORSS, H., SEPPÃ,,, L.	Prevention of enamel demineralization adjacent to glass ionomer filling materials	European Journal of Oral Sciences	Serch noise
2018	Fracasso, M.L.C., Venante, H.S., Santin, G.C., Salles, C.L.F., Provenzano, M.G.A., Maciel, S.M.	Performance of preventive methods applied to the occlusal surface of primary teeth: A randomized clinical study	Pesquisa Brasileira em Odontopediatria e Clinica Integrada	Different outcome
1966	Francis, M.D.	The effectiveness of anticaries agents in rats using an incipient carious lesion method	Archives of Oral Biology	Different type of study
1966	Francis, M.D., Briner, W.W.	The development and regression of hypomineralized areas of rat molars	Archives of Oral Biology	Different type of study
2020	Fransois, P., Greenwall-Cohen, J., Goff, S.L., Ruscassier, N., Attal, J.-P., Dursun, E.	Shear bond strength and interfacial analysis of high-viscosity glass ionomer cement bonded to dentin with protocols including silver diammine fluoride	Journal of Oral Science	Different outcome
2017	Frencken JE.	How useful is restorative care in the primary dentition?	Ned Tijdschr Tandheelkd	Different type of study
2020	Froehlich, T.T., Rocha, R.D.O., Botton, G.	Does previous application of silver diammine fluoride influence the bond strength of glass ionomer cement and adhesive systems to dentin? Systematic review and meta-analysis	International Journal of Paediatric Dentistry	Different type of study
1977	Gallagher, I.H.C., Cutress, T.W.	The effect of trace elements on the growth and fermentation by oral Streptococci and Actinomyces	Archives of Oral Biology	Serch noise
2021	Gao S.S., Zheng F.M., Chen K.J., Duangthip D., Lo E.C.M., Chu C.H.	Comparing two fluoride therapies for caries management in young children: study protocol for a randomised clinical trial	Trials	Different type of study
2016	Gao SS, Zhang S, Mei ML, Lo EC, Chu CH.	Caries remineralisation and arresting effect in children by professionally applied fluoride treatment - a systematic review	BMC Oral Health	Different type of study

2018	Gao SS, Zhao IS, Duffin S, Duangthip D, Lo ECM, Chu CH.	Revitalising Silver Nitrate for Caries Management	Int J Environ Res Public Health	Serch noise
2016	Gao SS, Zhao IS, Hiraishi N, Duangthip D, Mei ML, Lo ECM, Chu CH.	Clinical Trials of Silver Diamine Fluoride in Arresting Caries among Children: A Systematic Review	JDR Clin Trans Res	Different type of study
2019	Garg S., Sadr A., Chan D.	Potassium Iodide Reversal of Silver Diamine Fluoride Staining: A Case Report	Operative dentistry	Different outcome
2020	Garrastazu M.D., Mathias-Santamaria I.F., Rocha R.S., Diniz M.B., Caneppele T.M.F., Bresciani E.	Three-Month Effect of Silver Diamine Fluoride (SDF) in Salivary Levels of Streptococcus Mutans in Children. An Exploratory Trial	Oral health & preventive dentistry	Different outcome
2018	Gastemeyer G, Kohls A, Paris S, Schwendicke F.	Root caries prevention via sodium fluoride, chlorhexidine and silver diamine fluoride in vitro	Odontology	Serch noise
2017	Gastemeyer G, Schulze F, Paris S, Schwendicke F.	Arrest of Root Carious Lesions via Sodium Fluoride, Chlorhexidine and Silver Diamine Fluoride In Vitro	Materials (Basel)	Different outcome
2012	Geerts, S., Bolette, A., Seidel, L., Guaders, A.	An in vitro evaluation of leakage of two etch and rinse and two self-etch adhesives after thermocycling	International Journal of Dentistry	Serch noise
2001	Ghersel, E.L., Guedes-Pinto, A.C., Ciamponi, A.L.	Effect of the storage method on microleakage of primary teeth restored with different bonding systems: in vitro study	Pesquisa odontolÃ³gica brasileira = Brazilian oral research	Different type of study
2018	Giusti L, Steinborn C, Steinborn M.	Use of silver diamine fluoride for the maintenance of dental prostheses in a high caries-risk patient: A medical management approach	J Prosthet Dent	Different type of study
2001	Gladys, S., Van Meerbeek, B., Lambrechts, P., Vanherle, G.	Microleakage of adhesive restorative materials	American Journal of Dentistry	Serch noise
2013	Gluzman R, Katz RV, Frey BJ, McGowan R.	Prevention of root caries: a literature review of primary and secondary preventive agents	Spec Care Dentist	Serch noise

2019	Gold J.	Silver Diamine Fluoride May Prevent and Arrest Root Caries in Older Adults	J Evid Based Dent Pract	Different outcome
2018	Gold J.	Silver Diamine Fluoride Arrests Caries in Primary Teeth	J Evid Based Dent Pract	Different type of study
2020	Gold J.	Silver Diamine Fluoride Prevents Caries in Primary Teeth Superior to No Treatment, Placebo, or Fluoride Varnish	J Evid Based Dent Pract	Different type of study
2018	Gordon NB.	Silver Diamine Fluoride Staining is Acceptable for Posterior Primary Teeth and Is Preferred Over Advanced Pharmacologic Behavior Management by Many Parents	J Evid Based Dent Pract	Different outcome
1996	Gotjamanos T.	Pulp response in primary teeth with deep residual caries treated with silver fluoride and glass ionomer cement ('atraumatic' technique)	Australian dental journal	Different outcome
1997	Gotjamanos T.	Safety issues related to the use of silver fluoride in paediatric dentistry	Aust Dent J	Different type of study
2000	Gotjamanos, T., Ma, P.	Potential of 4 per cent silver fluoride to induce fluorosis in rats: Clinical implications	Australian Dental Journal	Different outcome
1998	Gotjamanos, T., Orton, V.	Abnormally high fluoride levels in commercial preparations of 40 per cent silver fluoride solution: Contraindications for use in children	Australian Dental Journal	Different outcome
2021	Grandjean ML, Maccarone NR, McKenna G, Maller F, Srinivasan M.	Silver Diamine Fluoride (SDF) in the management of root caries in elders: a systematic review and meta-analysis	Swiss Dent J	Different outcome
1989	Green, E.	A clinical evaluation of two methods of caries prevention in new-erupted first permanent molars	Australian Dental Journal	Different outcome
2020	Greenwall-Cohen J., Greenwall L., Barry S.	Silver diamine fluoride - an overview of the literature and current clinical techniques	British dental journal	Different type of study

2015	Gregory D, Hyde S.	Root Caries in Older Adults	J Calif Dent Assoc	Serch noise
2017	Gugnani N, Gugnani S.	Remineralisation and arresting caries in children with topical fluorides	Evid Based Dent	Different type of study
2021	Haiat A, Ngo HC, Samaranayake LP, Fakhruddin KS.	The effect of the combined use of silver diamine fluoride and potassium iodide in disrupting the plaque biofilm microbiome and alleviating tooth discoloration: A systematic review	PLoS One	Different type of study
1991	HALS, E.	Columna like structures of human dentin in carious and artificial lesions	European Journal of Oral Sciences	Different type of study
2015	Hamama, H.H., Yiu, C.K., Burrow, M.F.	Effect of silver diamine fluoride and potassium iodide on residual bacteria in dentinal tubules	Australian Dental Journal	Different outcome
2021	Hamdy D, Giraki M, Abd Elaziz A, Badran A, Allam G, Ruettermann S.	Laboratory evaluation of the potential masking of color changes produced by silver diamine fluoride in primary molars	BMC Oral Health	Different type of study
2020	Hammersmith K.J., DePalo J.R., Casamassimo P.S., MacLean J.K., Peng J.	Silver Diamine Fluoride and Fluoride Varnish May Halt Interproximal Caries Progression in the Primary Dentition	The Journal of clinical pediatric dentistry	Different type of sample
2019	Haque N, Widera D, Abu Kasim NH.	Stem Cells from Human Extracted Deciduous Teeth Expanded in Foetal Bovine and Human Sera Express Different Paracrine Factors After Exposure to Freshly Prepared Human Serum	Adv Exp Med Biol	Serch noise
1990	HARSTEDABINDSLEV, P., LARSEN, M.J.	Release of fluoride from conventional and metal-reinforced glass-ionomer cements	European Journal of Oral Sciences	Serch noise
2012	Hasegawa T, Chosa N, Asakawa T, Yoshimura Y, Fujihara Y, Kitamura T, Tanaka M, Ishisaki A, Mitome M.	Differential effects of TGF-beta 1 and FGF-2 on SDF-1 alfa expression in human periodontal ligament cells derived from deciduous teeth in vitro	Int J Mol Med	Serch noise
1989	Hattab, F.N., Mok, N.Y., Agnew, E.C.	Artificially formed carieslike lesions around restorative materials.	Journal of the American Dental Association (1939)	Different type of study

2015	Hayes M.	Topical agents for root caries prevention	Evid Based Dent	Different outcome
2008	Heintze, S., Forjanic, M., Cavalleri, A.	Microleakage of class II restorations with different tracers - Comparison with SEM quantitative analysis	Journal of Adhesive Dentistry	Serch noise
2017	Hendre AD, Taylor GW, ChÃ¡vez EM, Hyde S.	A systematic review of silver diamine fluoride: Effectiveness and application in older adults	Gerodontology	Different type of study
2020	Hiremath A.M., Anbu V., Kuduruthullah S., Khalil E., Elsahn N.A., Samuel S.R.	Acceptability of silver diamine fluoride as interim measure towards untreated dental caries and its impact on ohrqol among children with HIV: Pilot study	Indian journal of dental research : official publication of Indian Society for Dental Research	Different outcome
2013	Hooshmand, T., Tabari, N., Keshvad, A.	Marginal leakage and microhardness evaluation of low-shrinkage resin-based restorative materials	General Dentistry	Serch noise
2016	Horst JA, Ellenikiotis H, Milgrom PL.	UCSF Protocol for Caries Arrest Using Silver Diamine Fluoride: Rationale, Indications and Consent	J Calif Dent Assoc	Different type of study
2019	Horst JA, Heima M.	Prevention of Dental Caries by Silver Diamine Fluoride	Compend Contin Educ Dent	Different type of study
2018	Horst JA, Tanzer JM, Milgrom PM.	Fluorides and Other Preventive Strategies for a Tooth Decay	Dent Clin North Am	Different type of study
2018	Horst JA.	Silver Fluoride as a Treatment for Dental Caries	Adv Dent Res	Different type of study
1985	Hosoya Y.	Effect of acid etchants and restorative resin penetration into primary dentin after diammine silver fluoride application (I)	Shoni Shikagaku Zasshi	Different outcome
2010	Hosoya, Y., Ando, S., Yamaguchi, K., Oooka, S., Miyazaki, M., Tay, F.R.	Quality of the interface of primary tooth dentin bonded with antibacterial fluoride-releasing adhesive	Journal of Dentistry	Serch noise

2012	Hosoya, Y., Watanabe, E., Tadokoro, K., Inoue, T., Miyazaki, M., Tay, F.R.	Effects of ammonium hexafluorosilicate application on demineralized enamel and dentin of primary teeth.	Journal of oral science	Serch noise
2018	Hu S, Meyer B, Duggal M.	A silver renaissance in dentistry	Eur Arch Paediatr Dent	Different type of study
2020	Hu S., Meyer B., Lai B.W.P., Chay P.L., Tong H.J.	Parental acceptance of silver diammine fluoride in children with autism spectrum disorder	International journal of paediatric dentistry	Different outcome
2020	Huebner C.E., Milgrom P., Cunha-Cruz J., Scott J., Spiekerman C., Ludwig S., Mitchell M., Allen G., Dysert J., Shirtcliff R.M.	Parents' Satisfaction with Silver Diamine Fluoride Treatment of Carious Lesions in Children	Journal of dentistry for children (Chicago, Ill.)	Different outcome
2019	Ishiguro, T., Mayanagi, G., Azumi, M., Otani, H., Fukushima, A., Sasaki, K., Takahashi, N.	Sodium fluoride and silver diamine fluoride-coated tooth surfaces inhibit bacterial acid production at the bacteria/tooth interface	Journal of Dentistry	Different outcome
2020	Jabin Z, Vishnupriya V, Agarwal N, Nasim I, Jain M, Sharma A.	Effect of 38% silver diamine fluoride on control of dental caries in primary dentition: A Systematic review	J Family Med Prim Care	Different type of study
2021	Janakiram C, Ramanarayanan V, Devan I.	Effectiveness of Silver Diammine Fluoride Applications for Dental Caries Cessation in Tribal Preschool Children in India: Study Protocol for a Randomized Controlled Trial	Methods Protoc	Different type of study
2021	Jasulaityte L, Burgersdijk RCW.	Non-restorative cavity treatment: from guidelines to the practice	Ned Tijdschr Tandheelkd	Different type of study
2020	Jiang M, Mei ML, Wong M, Chu CH, Lo E.	Influence of Silver Diamine Fluoride Treatment on the Microtensile Bond Strength of Glass Ionomer Cement to Sound and Carious Dentin	Oper Dent	Different outcome
2019	Jiang M., Wong M.C.M., Chu C.H., Dai L., Lo E.C.M.	Effects of restoring SDF-treated and untreated dentine caries lesions on parental satisfaction and oral health related quality of life of preschool children	Journal of dentistry	Different outcome
2020	Jiang M., Wong M.C.M., Chu C.H., Dai L., Lo E.C.M.	A 24-month randomized controlled trial on the success rates of restoring untreated and SDF-treated dentine caries lesions in primary teeth with the ART approach	Journal of dentistry	Different outcome

2021	Jiang M., Xie Q.Y., Wong M.C.M., Chu C.H., Lo E.C.M.	Association between dental conditions, sliver diamine fluoride application, parental satisfaction, and oral health-related quality of life of preschool children	Clinical oral investigations	Different outcome
2021	Jiang, C.M., Duangthip, D., Chan, A.K.Y., Tamrakar, M., Lo, E.C.M., Chu, C.H.	Global research interest regarding silver diamine fluoride in dentistry: A bibliometric analysis	Journal of Dentistry	Different type of study
2012	Jiang, L., Peng, W.-W., Li, L.-F., Yang, Y., Zhu, Y.-Q.	Isolation and identification of cxcr4-positive cells from human dental pulp cells	Journal of Endodontics	Serch noise
2019	Johhnson B., Serban N., Griffin P.M., Tomar S.L.	Projecting the economic impact of silver diamine fluoride on caries treatment expenditures and outcomes in young U.S. children	Journal of public health dentistry	Different outcome
2019	Karched M, Ali D, Ngo H.	In vivo antimicrobial activity of silver diammine fluoride on carious lesions in dentin	J Oral Sci	Different outcome
2005	Kawasaki A, Suge T, Ishikawa K, Ozaki K, Matsuo T, Ebisu S.	Ammonium hexafluorosilicate increased acid resistance of bovine enamel and dentine	J Mater Sci Mater Med	Different outcome
2018	Khoroushi, M., Etemadi, S., Kheir, M.	Marginal Leakage of Class v Composite Resin Restorations	Dental Hypotheses	Serch noise
2016	Kim JH, Kim GH, Kim JW, Pyeon HJ, Lee JC, Lee G, Nam H.	In Vivo Angiogenic Capacity of Stem Cells from Human Exfoliated Deciduous Teeth with Human Umbilical Vein Endothelial Cells	Mol Cells	Serch noise
2021	Kim S, Nassar M, Tamura Y, Hiraishi N, Jamleh A, Nikaido T, Tagami J.	The effect of reduced glutathione on the toxicity of silver diamine fluoride in rat pulpal cells	J Appl Oral Sci	Different outcome
2016	Kirschneck, C., Christl, J.-J., Reicheneder, C., Proff, P.	Efficacy of fluoride varnish for preventing white spot lesions and gingivitis during orthodontic treatment with fixed appliances: a prospective randomized controlled trial	Clinical Oral Investigations	Serch noise
1999	Klein U, Kanellis MJ, Drake D.	Effects of four anticaries agents on lesion depth progression in an in vitro caries model	Pediatr Dent	Different type of study

2010	Knight GM, McIntyre JM, Craig GG, Mulyani.	Leave decay in my cavity? You must be kidding!	Dent Today	Serch noise
2006	Knight, G.M., McIntyre, J.M., Craig, G.G., Mulyani	Ion uptake into demineralized dentine from glass ionomer cement following pretreatment with silver fluoride and potassium iodide	Australian Dental Journal	Wrong study design
2007	Knight, G.M., McIntyre, J.M., Craig, G.G., Mulyani, Zilm, P.S., Gully, N.J.	Differences between normal and demineralized dentine pretreated with silver fluoride and potassium iodide after an in vitro challenge by Streptococcus mutans	Australian Dental Journal	Different outcome
2009	Knight, G.M., McIntyre, J.M., Craig, G.G., Mulyani, Zilm, P.S., Gully, N.J.	Inability to form a biofilm of Streptococcus mutans on silver fluoride- and potassium iodide-treated demineralized dentin	Quintessence International	Different outcome
2005	Knight, G.M., McIntyre, J.M., Craig, G.G., Mulyani, Zilm, P.S., Gully, N.J.	An in vitro model to measure the effect of a silver fluoride and potassium iodide treatment on the permeability of demineralized dentine to Streptococcus mutans	Australian Dental Journal	Wrong study design
2006	Knight, G.M., McIntyre, J.M., Mulyani	The effect of silver fluoride and potassium iodide on the bond strength of auto cure glass ionomer cement to dentine	Australian Dental Journal	Different outcome
2013	Kolb, A.K., Schmied, K., FaÃŸheber, P., Heinrich-Weltzien, R.	Preschool children's taste acceptance of highly concentrated fluoride compounds: Effects on nonverbal behavior	Journal of Clinical Pediatric Dentistry	Serch noise
2021	Kopczynski K., Meyer B.D.	Examining parental treatment decisions within a contemporary pediatric dentistry private practice	Patient Preference and Adherence	Serch noise
2019	Kuang, B., Zeng, Z., Qin, Q.	Biomechanically stimulated chondrocytes promote osteoclastic bone resorption in the mandibular condyle	Archives of Oral Biology	Serch noise
2002	Kubo, S., Li, H., Burrow, M.F., Tyas, M.J.	Nanoleakage of dentin adhesive systems bonded to carisolv-treated dentin	Operative Dentistry	Serch noise
1989	Kubota M.	Insolubilization of deciduous tooth enamel by the two-step method with fluoride and lanthanum treatment	Kanagawa Shigaku	Different type of study

2016	Kucukyilmaz E, Savas S, Akcay M, Bolukbasi B.	Effect of silver diamine fluoride and ammonium hexafluorosilicate applications with and without Er:YAG laser irradiation on the microtensile bond strength in sound and caries-affected dentin	Lasers Surg Med	Different type of study
2019	Kumar A., Cernigliaro D., Northridge M.E., Wu Y., Troxel A.B., Cunha-Cruz J., Balzer J., Okuji D.M.	A survey of caregiver acculturation and acceptance of silver diamine fluoride treatment for childhood caries	BMC oral health	Different outcome
1993	Kupietzky, A.	Teaching kindergarten and elementary school children dental health: a practical presentation.	The Journal of clinical pediatric dentistry	Serch noise
2020	Kurkina, O.N., Osokina, A.S., Makedonova, Y.A., Afonina, I.V., Pyshnenko, V.R., Kurkina, V.M., Maslak, E.E.	Parents' compliance with silver diamine fluoride use for treatment of caries lesions in children	Journal of International Dental and Medical Research	Different outcome
2020	Kyoon-Achan G., Schroth R.J., DeMarÃ© D., Sturym M., Edwards J., Lavoie J.G., Sanguins J., Campbell R., Chartrand F., Bertone M.F., Singh S., Moffatt M.E.K.	Indigenous community members' views on silver diamine fluoride to manage early childhood caries	Journal of public health dentistry	Different outcome
2021	Kyoon-Achan G., Schroth R.J., Martin H., Bertone M., Mittermuller B.A., Sihra R., Klus B., Singh S., Moffatt M.E.K.	Parents' Views on Silver Diamine Fluoride to Manage Early Childhood Caries	JDR clinical and translational research	Different outcome
2021	Lau L, Quock RL, Wu DI, Harrington DA, Patel SA, Barros JA.	Effect of surface preparation and light curing on penetration of silver particles from 38% silver diamine fluoride in dentin of primary teeth: An in vitro evaluation	Am J Dent	Wrong study design
2013	Laupez, R.M., Uribe, M.R., RodrÃguez, B.O., Casasempere, I.V.	Comparison between amine fluoride and chlorhexidine with institutionalized elders: A pilot study	Gerodontology	Different type of sample
2014	Lenzi, T.L., Braga, M.M., Raggio, D.P.	Shortening the etching time for etch-and-rinse adhesives increases the bond stability to simulated caries-affected primary dentin	Journal of Adhesive Dentistry	Serch noise
2015	Lenzi, T.L., Raggio, D.P., Soares, F.Z.M., Rocha, R.O.	Bonding performance of a multimode adhesive to artificially-induced caries-affected primary dentin	Journal of Adhesive Dentistry	Serch noise
2015	Lewis A, Wallace J, Deutsch A, King P.	Improving the oral health of frail and functionally dependent elderly	Aust Dent J	Serch noise

2016	Li R, Lo EC, Liu BY, Wong MC, Chu CH.	Randomized clinical trial on arresting dental root caries through silver diammine fluoride applications in community-dwelling elders	J Dent	Different outcome
2017	Li R, Lo ECM, Liu BY, Wong MCM, Chu CH.	Randomized Clinical Trial on Preventing Root Caries among Community-Dwelling Elders	JDR Clin Trans Res	Different outcome
2019	Li Y, Liu Y, Psoter WJ, Nguyen OM, Bromage TG, Walters MA, Hu B, Rabieh S, Kumararaja FC.	Assessment of the Silver Penetration and Distribution in Carious Lesions of Deciduous Teeth Treated with Silver Diamine Fluoride	Caries Res	Different outcome
1984	Li YJ.	Effect of a silver ammonia fluoride solution on the prevention and inhibition of caries	Zhonghua Kou Qiang Ke Za Zhi	Serch noise
2014	Li, D., Fu, L., Zhang, Y., Yu, Q., Ma, F., Wang, Z., Luo, Z., Zhou, Z., Cooper, P.R., He, W.	The effects of LPS on adhesion and migration of human dental pulp stem cells in vitro	Journal of Dentistry	Serch noise
2002	Li, H., Burrow, M.F., Tyas, M.J.	The effect of load cycling on the nanoleakage of dentin bonding systems	Dental Materials	Serch noise
2002	Li, H., Burrow, M.F., Tyas, M.J.	The effect of thermocycling regimens on the nanoleakage of dentin bonding systems	Dental Materials	Serch noise
2021	Lim, G.X.D., Yang, J.	Effect of silver diamine fluoride on hyperplastic gingivitis in an adult with intellectual disabilityâ€"A case report	Special Care in Dentistry	Different outcome
2019	Lin YS, Rothen ML, Milgrom P.	Pharmacokinetics of 38% topical silver diamine fluoride in healthy adult volunteers	J Am Dent Assoc	Different outcome
2020	Liu B.Y., Liu J., Zhang D., Yang Z.L., Feng Y.P., Wang M.	Effect of silver diammine fluoride on micro-ecology of plaque from extensive caries of deciduous teeth - in vitro study	BMC oral health	Different type of study
2012	Liu B.Y., Lo E.C.M., Chu C.H., Lin H.C.	Randomized trial on fluorides and sealants for fissure caries prevention	Journal of Dental Research	Different outcome

2019	Liu BY, Mei L, Chu CH, Lo ECM.	Effect of Silver Fluoride in Preventing the Formation of Artificial Dentinal Caries Lesions in vitro	Chin J Dent Res	Different type of study
2001	Lo EC, Chu CH, Lin HC.	A community-based caries control program for pre-school children using topical fluorides: 18-month results	J Dent Res	Different outcome
2013	Lopes Coutinho, T.C., Almeida Tostes, M.	Comparison of microleakage of different margin types around Class V resin restorations in primary teeth	European Journal of Paediatric Dentistry	Serch noise
2018	Lou Y, Darvell BW, Botelho MG.	Antibacterial Effect of Silver Diammine Fluoride on Cariogenic Organisms	J Contemp Dent Pract	Different outcome
2011	Lou, Y.L., Botelho, M.G., Darvell, B.W.	Reaction of silver diamine fluoride with hydroxyapatite and protein	Journal of Dentistry	Different type of study
2020	Luk K, Zhao IS, Yu OY, Mei ML, Gutknecht N, Chu CH.	Caries Prevention Effects of Silver Diamine Fluoride with 10,600 nm Carbon Dioxide Laser Irradiation on Dentin	Photobiomodul Photomed Laser Surg	Different outcome
2017	Magalhaes AC.	Conventional Preventive Therapies (Fluoride) on Root Caries Lesions	Monogr Oral Sci	Different outcome
2019	Magno MB, Silva LPD, Ferreira DM, Barja-Fidalgo F, Fonseca-GonÃ§alves A.	Aesthetic perception, acceptability and satisfaction in the treatment of caries lesions with silver diamine fluoride: A scoping review	Int J Paediatr Dent	Different type of study
2020	Markham MD, Tsujimoto A, Barkmeier WW, Jurado CA, Fischer NG, Watanabe H, Baruth AG, Latta MA, Garcia-Godoy F.	Influence of 38% silver diamine fluoride application on bond stability to enamel and dentin using universal adhesives in self-etch mode	Eur J Oral Sci	Different outcome
1997	Martin AP.	Silver fluoride use	Aust Dent J	Different type of study
2015	Mattos-Silveira J., Floriano I., Ferreira F.R., Vigana M.E., Mendes F.M., Braga M.M.	Children's discomfort may vary among different treatments for initial approximal caries lesions: preliminary findings of a randomized controlled clinical trial	International journal of paediatric dentistry	Different outcome

2014	Mattos-Silveira J., Floriano I., Ferreira F.R., Vigana M.E.F., Frizzo M.A., Reyes A., Novaes T.F., Moriyama C.M., Raggio D.P., Imparato J.C.P., Mendes F.M., Braga M.M.	New proposal of silver diamine fluoride use in arresting approximal caries: Study protocol for a randomized controlled trial	Trials	Different type of sample
2020	McComas MJ, Hurlbutt M, Fontana M.	A survey of cariology education in U.S. dental hygiene programs: The need for a core curriculum framework	J Dent Educ	Serch noise
2018	McReynolds D, Duane B.	Systematic review finds that silver diamine fluoride is effective for both root caries prevention and arrest in older adults	Evid Based Dent	Different type of study
2014	Mei M.L., Ito L., Cao Y., Lo E.C., Li Q.L., Chu C.H.	An ex vivo study of arrested primary teeth caries with silver diamine fluoride therapy	Journal of dentistry	Different type of study
2020	Mei M.L., Yan Z., Duangthip D., Niu J.Y., Yu O.Y., You M., Lo E.C.M., Chu C.H.	Effect of silver diamine fluoride on plaque microbiome in children	Journal of dentistry	Different outcome
2013	Mei ML, Chu CH, Low KH, Che CM, Lo EC.	Caries arresting effect of silver diamine fluoride on dentine carious lesion with S. mutans and L. acidophilus dual-species cariogenic biofilm	Med Oral Patol Oral Cir Bucal	Different outcome
2014	Mei ML, Ito L, Chu CH, Lo EC, Zhang CF.	Prevention of dentine caries using silver diamine fluoride application followed by Er:YAG laser irradiation: an in vitro study	Lasers Med Sci	Different type of study
2015	Mei ML, Ito L, Zhang CF, Lo EC, Chu CH.	Effect of laser irradiation on the fluoride uptake of silver diamine fluoride treated dentine	Lasers Med Sci	Different outcome
2013	Mei ML, Li QL, Chu CH, Lo EC, Samaranayake LP.	Antibacterial effects of silver diamine fluoride on multi-species cariogenic biofilm on caries	Ann Clin Microbiol Antimicrob	Wrong study design
2016	Mei ML, Lo EC, Chu CH.	Clinical Use of Silver Diamine Fluoride in Dental Treatment	Compend Contin Educ Dent	Different type of study
2018	Mei ML, Lo ECM, Chu CH.	Arresting Dentine Caries with Silver Diamine Fluoride: What's Behind It?	J Dent Res	Different type of study

2017	Mei ML, Nudelman F, Marzec B, Walker JM, Lo ECM, Walls AW, Chu CH.	Formation of Fluorohydroxyapatite with Silver Diamine Fluoride	J Dent Res	Different outcome
2016	Mei ML, Zhao IS, Ito L, Lo EC, Chu CH.	Prevention of secondary caries by silver diamine fluoride	Int Dent J	Different outcome
2013	Mei, M.L., Chu, C.H., Lo, E.C.M., Samaranayake, L.P.	Fluoride and silver concentrations of silver diammine fluoride solutions for dental use	International Journal of Paediatric Dentistry	Different outcome
2014	Mei, M.L., Ito, L., Cao, Y., Li, Q.L., Chu, C.H., Lo, E.C.M.	The inhibitory effects of silver diamine fluorides on cysteine cathepsins	Journal of Dentistry	Different outcome
2012	Mei, M.L., Li, Q.L., Chu, C.H., Yiu, C.K.Y., Lo, E.C.M.	The inhibitory effects of silver diamine fluoride at different concentrations on matrix metalloproteinases	Dental Materials	Different outcome
2021	Mendiratta M, B C M, Kumar A, Yadav V, Shyam R, Wig M.	Effectiveness of silver diamine fluoride and glass ionomer cement combined with fluoride varnish in arresting dental caries among intellectually disabled individuals: A randomized controlled trial	Spec Care Dentist	Different outcome
2019	Meyer-Lueckel H, Machiulskiene V, Giacaman RA.	How to Intervene in the Root Caries Process? Systematic Review and Meta-Analyses	Caries Res	Different type of study
2009	Milgrom P, Zero DT, Tanzer JM.	An examination of the advances in science and technology of prevention of tooth decay in young children since the Surgeon General's Report on Oral Health	Acad Pediatr	Different outcome
2011	Milgrom P., Chi D.L.	Prevention-centered caries management strategies during critical periods in early childhood.	Journal of the California Dental Association	Different outcome
2019	Miller DB.	A new protocol and standard of care for managing open crown margins	Gen Dent	Different type of study
2016	Miller MB, Laupez LA, Quock RL.	Silver diamine fluoride, potassium iodide, and esthetic perception: An in vitro pilot study	Am J Dent	Wrong study design

2021	Minavi B, Youssefi A, Quock R, Letra A, Silva R, Kirkpatrick TC, Tribble G, van der Hoeven R.	Evaluating the substantivity of silver diamine fluoride in a dentin model	Clin Exp Dent Res	Different outcome
2020	Mishra S., Mohanty S.	Silver diamine fluoride in clinical pediatric dentistry	Indian Journal of Forensic Medicine and Toxicology	Different type of study
2021	Mitchell C, Gross AJ, Milgrom P, Mancl L, Prince DB.	Silver diamine fluoride treatment of active root caries lesions in older adults: A case series	J Dent	Different outcome
2021	Mitchell C, Zaku H, Milgrom P, Mancl L, Prince DB.	The accuracy of laser fluorescence (DIAGNOdent) in assessing caries lesion activity on root surfaces, around crown margins, and in furcations in older adults	BDJ Open	Serch noise
2019	Mitwalli H, Mourao MDA, Dennison J, Yaman P, Paster BJ, Fontana M.	Effect of Silver Diamine Fluoride Treatment on Microbial Profiles of Plaque Biofilms from Root/Cervical Caries Lesions	Caries Res	Different outcome
2018	Mohammadi N, Farahmand Far MH.	Effect of fluoridated varnish and silver diamine fluoride on enamel demineralization resistance in primary dentition	J Indian Soc Pedod Prev Dent	Different outcome
2012	Monse B, Heinrich-Weltzien R, Mulder J, Holmgren C, van Palenstein Helderman WH.	Caries preventive efficacy of silver diammine fluoride (SDF) and ART sealants in a school-based daily fluoride toothbrushing program in the Philippines	BMC Oral Health	Different outcome
2021	Moradi S., Sabbagh S., Timms L., Ravaghi V.	Teaching Minimally Invasive Interventions in Paediatric Dentistry: A Cross-Sectional Survey of Dental Schools in Iran	BMC oral health	Different outcome
2021	Moreira, K.M., Bertassoni, L.E., Davies, R.P., Joia, F., Hofling, J.F., Nascimento, F.D., Puppin-Rontani, R.M.	Impact of biomineralization on resin/biomineralized dentin bond longevity in a minimally invasive approach: An â€œin vitroâ€ 18-month follow-up	Dental Materials	Serch noise
1974	Moriwaki Y, Shimizu A, Nonomura E, Sobue S, Tsutsumi S.	X-ray microbeam diffraction studies on the effect of diamine silver fluoride	Shika Rikogaku Zasshi	Abstract or full-text not avable
1996	Murata H, Ishikawa K, Tenshin S, Horiuchi S, Nakanishi M, Asaoka K, Kawata T, Yamamoto TT.	Fluoridation of hydroxyapatite powder by ammonium hexafluorosilicate	Caries Res	Serch noise

1998	Naasan, M.A., Watson, T.F.	Conventional glass ionomers as posterior restorations: A status report for the American Journal of Dentistry	American Journal of Dentistry	Serch noise
2021	Nainar S.M.H.	Silver Diamine Fluoride Usage in Children	Pediatric dentistry	Abstract or full-text not avable
2016	Nantanee R, Santiwong B, Trairatvorakul C, Hamba H, Tagami J.	Silver diamine fluoride and glass ionomer differentially remineralize early caries lesions, in situ	Clin Oral Investig	Different outcome
2012	Neelakantan, P., Rao, C.V.S., Indramohan, J.	Bacteriology of deep carious lesions underneath amalgam restorations with different pulp-capping materials - An in vivo analysis	Journal of Applied Oral Science	Serch noise
2020	Nelson S, Albert JM, Milgrom P.	Comparative Effectiveness of Two Nonsurgical Treatments to Reduce Oral Health Disparities From Untreated Tooth Decay in Older Adults: Protocol for a Cluster Randomized Trial	JMIR Res Protoc	Different type of sample
2016	Nelson T., Scott J.M., Crystal Y.O., Berg J.H., Milgrom P.	Silver Diamine Fluoride in Pediatric Dentistry Training Programs: Survey of Graduate Program Directors	Pediatric dentistry	Different outcome
2020	Ng E, Saini S, Schulze KA, Horst J, Le T, Habelitz S.	Shear Bond Strength of Glass Ionomer Cement to Silver Diamine Fluoride-Treated Artificial Dentinal Caries	Pediatr Dent	Different type of study
2018	Ngoc CN, Mehta R, Donovan TE, Ferreira Zandona AG.	Teaching Silver Diamine Fluoride in U.S. Dental Schools' Predoctoral Curricula	J Dent Educ	Different outcome
1981	Nielsen A., Ravn J.J.	Treatment with silver nitrate in pedodontics	Tandlaegebladet	Abstract or full-text not avable
1977	Nishino M, Massler M.	Immunization of caries-susceptible pits and fissures with a diammine silver fluoride solution	The Journal of pedodontics	Abstract or full-text not avable
1974	Nishino M, Ono S, Kita Y, Tsuchitani Y.	Caries prevention in pits and fissures with diammine silver fluoride solution and fissure sealant. Sealing properties of pits and fissures and adhesive characteristics to enamel	J Osaka Univ Dent Sch	Different outcome

1969	Nishino M, Yoshida S, Sobue S, Kato J, Nishida M.	Effect of topically applied ammoniacal silver fluoride on dental caries in children	J Osaka Univ Dent Sch	Abstract or full-text not avable
1969	Nishino M.	Studies on the topical application of ammoniacal silver fluoride for the arrest of dental caries	Osaka Daigaku Shigaku Zasshi	Abstract or full-text not avable
2020	Nizami MZI, Nishina Y, Yamamoto T, Shinoda-Ito Y, Takashiba S.	Functionalized Graphene Oxide Shields Tooth Dentin from Decalcification	J Dent Res	Serch noise
2017	No authors listed	Chairside Guide: Silver Diamine Fluoride in the Management of Dental Caries Lesions	Pediatric dentistry	Different type of study
2017	No authors listed	Limited evidence suggesting silver diamine fluoride may arrest dental caries in children	British dental journal	Different type of study
2017	Nozari A, Ajami S, Rafiei A, Niazi E.	Impact of Nano Hydroxyapatite, Nano Silver Fluoride and Sodium Fluoride Varnish on Primary Teeth Enamel Remineralization: An In Vitro Study	J Clin Diagn Res	Serch noise
2020	Nurrohman H, Habelitz S, Saeki K, Sadr A, Gower LB, Pazdernik V, Tagami J, Marshall SJ, Marshall GW.	Enhanced silver diamine fluoride therapy using the PILP method -A nanoindentation study	Dent Mater J	Different outcome
1989	Nystrom, G.P., Holtan, J.R., Olin, P.S., Douglas, W.H.	Technical note: Fluoride pre-treatment effects on microleakage of a resin bonding agent	Dental Materials	Serch noise
1974	Okuyama T.	On the penetration of diammine silver fluoride into the carious dentin of deciduous teeth (author's transl)	Shigaku	Different outcome
2019	Oliveira B.H., Rajendra A., Veitz-Keenan A., Niederman R.	The Effect of Silver Diamine Fluoride in Preventing Caries in the Primary Dentition: A Systematic Review and Meta-Analysis	Caries research	Different type of study
2018	Oliveira BH, Cunha-Cruz J, Rajendra A, Niederman R.	Controlling caries in exposed root surfaces with silver diamine fluoride: A systematic review with meta-analysis	J Am Dent Assoc	Different outcome

2018	PÃ©rez-HernÃ¡ndez, J., Aguilar-DÃaz, F.C., Venegas-LancÃ³n, R.D., Gayosso, C.A.Ã ., Villanueva-Vilchis, M.C., de la Fuente-HernÃ¡ndez, J.	Effect of silver diamine fluoride on adhesion and microleakage of a pit and fissure sealant to tooth enamel: in vitro trial	European Archives of Paediatric Dentistry	Different type of study
2016	Paglia L.	Pit and fissure sealants or fluoride varnishes?	European journal of paediatric dentistry : official journal of European Academy of Paediatric Dentistry	Serch noise
2018	Pakdaman A, Montazeri A, Evans RW.	Deciduous dentition approximal caries lesion progression and regression following preventive treatment: literature review	Aust Dent J	Different type of study
2021	Panahpour Eslami N, Chan DCN, Sadr A.	Effect of silver diammine fluoride and glass ionomer on remineralisation of natural dentine caries	J Dent	Different type of study
2018	Patel J, Anthonappa RP, King NM.	Evaluation of the staining potential of silver diamine fluoride: in vitro	Int J Paediatr Dent	Different type of study
2021	Patel J, Foster D, Smirk M, Turton B, Anthonappa R.	Acidity, fluoride and silver ion concentrations in silver diamine fluoride solutions: a pilot study	Aust Dent J	Different outcome
2021	Paul B, Sierra MA, Xu F, Crystal YO, Li X, Saxena D, Ruff RR.	Microbial population shift and metabolic characterization of silver diamine fluoride treatment failure on dental caries	PLoS One	Different outcome
2018	Pedram, P., Hooshmand, T., Heidari, S.	Effect of different cavity lining techniques on marginal sealing of class II resin composite restorations in vitro	International Journal of Periodontics and Restorative Dentistry	Different type of study
2012	Peng JJ, Botelho MG, Matinlinna JP.	Silver compounds used in dentistry for caries management: a review	J Dent	Different type of study
2019	Peng JY, Tsoi JKH, Matinlinna JP, Botelho MG.	Silver deposition on demineralized dentine surface dosed by silver diammine fluoride with different saliva	J Investig Clin Dent	Different outcome

2003	Peris, A.R., Duarte Jr., S., De Andrade, M.F.	Evaluation of marginal microleakage in Class II cavities: Effect of microhybrid, flowable, and compactable resins	Quintessence International	Serch noise
2011	Pimentel, A.C., dos Santos, M.P.A., Maia, L.C., Dias, K.R.H.C.	Effect of enamel bevel on microleakage in class I cavities in primary molars	Pesquisa Brasileira em Odontopediatria e Clinica Integrada	Serch noise
2021	Piovesan ET, Silva MV, de Campos TA, Martins VD, Bezzerra ACB.	Antimicrobial effects of silver diamine fluoride: An in vivo study	Am J Dent	Different type of study
2021	Piovesan ET., Silva M.V., de Campos T.A., Martins V.D., Bezzerra A.C.B.	Effectiveness of two desensitizing products: A 6-month randomized clinical, split-mouth study	American journal of dentistry	Different outcome
2020	Pisarnturakit P., Detsomboonrat P.	Comparison of two caries prevention programs among Thai kindergarten: a randomized controlled trial	BMC oral health	Different outcome
2021	Punhagui MF, Jussiani EI, Andrello AC, Favaro JC, Guiraldo RD, Lopes MB, Berger SB.	Effect of application time and concentration of silver diamine fluoride on the enamel remineralization	J Clin Exp Dent	Different type of study
2018	Punyanirun K, Yospiboonwong T, Kunapinun T, Thanyasrisung P, Trairatvorakul C.	Silver diamine fluoride remineralized artificial incipient caries in permanent teeth after bacterial pH-cycling in-vitro	J Dent	Different outcome
2018	Puwanawiroj A, Trairatvorakul C, Dasanayake AP, Auychai P.	Microtensile Bond Strength Between Glass Ionomer Cement and Silver Diamine Fluoride-Treated Carious Primary Dentin	Pediatr Dent	Different type of study
2011	Quock RL, Patel SA, Falcao FA, Barros JA.	Is a drill-less dental filling possible?	Med Hypotheses	Serch noise
2012	Quock, R.L., Barros, J.A., Yang, S.W., Patel, S.A.	Effect of silver diamine fluoride on microtensile bond strength to dentin	Operative Dentistry	Different outcome
2017	Rajendra A., Veitz-Keenan A., Oliveira B.H., Ruff R.R., Wong M.C.M., Innes N.P.T., Radford J., Seifo N., Niederman R.	Topical silver diamine fluoride for managing dental caries in children and adults	Cochrane Database of Systematic Reviews	different type of study

2020	Rams TE, Sautter JD, RamÃrez-MartÃnez GJ, Whitaker EJ.	Antimicrobial activity of silver diamine fluoride on human periodontitis microbiota	Gen Dent	Different outcome
2012	Reis A., Chibinski A.C., Stanislawczuk R., Wambier D.S., Grande R.H., Loguercio A.D.	The role of dentin moisture in the degradation of resin-dentin interfaces under clinical and laboratory conditions.	Journal of the American Dental Association (1939)	Serch noise
2021	Renugalakshmi A., Vinothkumar T.S., Hakami F.B., Salem R.M., Qadri A.A., Harbosh Z.M., Hakami Z.	Impact of Silver Diamine Fluoride Therapy on Oral Health-related Quality of Life of Uncooperative Preschool Children: A Prospective Study	Oral health & preventive dentistry	Different outcome
2017	Richards D.	The effectiveness of silver diamine fluoride in arresting caries	Evid Based Dent	Different type of study
2020	Rizzante, F.A.P., Sedky, R.A.F., Furuse, A.Y., Teich, S., Ishikiriama, S.K., MendonÃ§a, G.	Validation of a method of quantifying 3D leakage in dental restorations	Journal of Prosthetic Dentistry	Serch noise
2020	Roberts A, Bradley J, Merkley S, Pachal T, Gopal JV, Sharma D.	Does potassium iodide application following silver diamine fluoride reduce staining of tooth? A systematic review	Aust Dent J	Different type of study
2019	Roberts-Thomson K.F., Ha D.H., Wooley S., Meihubers S., Do L.G.	Community trial of silver fluoride treatment for deciduous dentition caries in remote Indigenous communities	Australian dental journal	Search noise
2020	Rodrigues GF, Costa TDC, Massa GDS, Vollu AL, Barja-Fidalgo F, Fonseca-Goncalves A.	Oral Health-Related Quality of Life in Preschool Children After Silver Diamine Fluoride Versus Atraumatic Restorative Treatments	Pediatr Dent	Different outcome
2020	Romero DA, FernÃ¡ndez CE, de Melo Santos L.	Commercial Silver Diamine Fluoride (SDF) Products on Caries Lesion Progression in Primary Enamel: An In Vitro Study	Oral Health Prev Dent	Different type of study
2021	Romero MJRH, Lippert F.	Indirect caries-preventive effect of silver diamine fluoride on adjacent dental substrate: A single-section demineralization study	Eur J Oral Sci	Different type of study
2009	Rosenblatt A, Stamford TC, Niederman R.	Silver diamine fluoride: a caries "silver-fluoride bullet"	J Dent Res	Different type of study

2020	Roshni R.S., Shetty P.J.	Nano silver fluoride for arresting dental caries	Journal of Pharmaceutical Sciences and Research	Serch noise
2017	Rossi G, Squassi A, Mandalunis P, Kaplan A.	Effect of silver diamine fluoride (SDF) on the dentin-pulp complex: ex vivo histological analysis on human primary teeth and rat molars	Acta Odontol Latinoam	Different type of study
1991	Roth, S.	A laboratory study of glass ionomer cement as a retrograde rooth filling material	Australian Dental Journal	Different type of study
2019	Rubin M.S., Nunez N., Quick J.D., Edelstein B.L.	A survey of US early childhood caries programs: findings and recommendations	Journal of public health dentistry	Different type of study
2019	Rudiak, A., Opydo-Szymaczek, J., Opydo, W.	Fluoride content in drinking water from shallow dug wells in the agricultural area of subcarpathian province (Poland)	Fluoride	Serch noise
2018	Ruff R.R., Niederman R.	Comparative effectiveness of treatments to prevent dental caries given to rural children in school-based settings: Protocol for a cluster randomised controlled trial	BMJ Open	Different type of study
2018	Ruff RR, Niederman R.	Silver diamine fluoride versus therapeutic sealants for the arrest and prevention of dental caries in low-income minority children: study protocol for a cluster randomized controlled trial	Trials	Different type of study
1987	Russo M., Komatsu J., Takayama S., Holland JÃºnior C., Sundfeld R.H., Mestrener S.R., de Castro M.A., Quintella L.P.	Silver diamine fluoride. Pulp response to application of a 10% solution to dentin	RGO	Different outcome
2020	Sabbagh H., Othman M., Khogeer L., Al-Harbi H., Al Harthi A., Abdulgader Yaseen Abdulgader A.	Parental acceptance of silver Diamine fluoride application on primary dentition: a systematic review and meta-analysis	BMC oral health	Different outcome
2021	Salamoon M.A., Waly N.G., Elmasry E.S., Abdelgawad F.	Effect of discoloration of silver diamine fluoride versus sodium fluoride varnish in treatment of carious primary teeth: A randomized clinical trial	Indian Journal of Public Health Research and Development	Different outcome
2020	Sanjari, K., Bayani, M., Zadeh, H.E.	Conservative dental management of a patient with Epidermolysis bullosa. A case report	Pediatric Dental Journal	Different type of study

2009	Santos Lde M, Reis JI, Medeiros MP, Ramos SM, AraÃºjo JM.	In vitro evaluation of fluoride products in the development of carious lesions in deciduous teeth	Braz Oral Res	Different type of study
2014	Santos V.E., Vasconcelos Filho A., Targino A.G., Flores M.A., Galembeck A., Caldas A.F., Rosenblatt A.	A new "silver-bullet" to treat caries in children--nano silver fluoride: a randomised clinical trial	Journal of dentistry	Serch noise
1989	Sato M, Ozawa Y, Masaya M, Uchikawa Y, Tosaka S, Okumura T.	Clinical evaluation of the GaALAs laser treatment for hypersensitive dentin	Shigaku	Serch noise
2017	Satyanegara, A., Darwita, R.R., Setiawati, F., Adiatman, M., Muhammad, R.	An invitro study of caries arresting effect of propolis fluoride and silver diamine fluoride on dentine carious lesions	Journal of International Dental and Medical Research	Different type of study
2015	Savas S, KucukyÄ±lmaz E, Celik EU, Ates M.	Effects of different antibacterial agents on enamel in a biofilm caries model	J Oral Sci	Different type of study
2016	Savas S, Kucukyilmaz E, Celik EU.	Effects of Remineralization Agents on Artificial Carious Lesions	Pediatr Dent	Different type of study
2020	Sayed M, Hiraishi N, Matin K, Abdou A, Burrow MF, Tagami J.	Effect of silver-containing agents on the ultra-structural morphology of dentinal collagen	Dent Mater	Serch noise
2021	Sayed M, Nikaido T, Abdou A, Burrow MF, Tagami J.	Potential use of silver diammine fluoride in detection of carious dentin	Dent Mater J	Different type of study
2020	Schmoeckel J, Gorseta K, Splieth CH, Juric H.	How to Intervene in the Caries Process: Early Childhood Caries - A Systematic Review	Caries Res	Different type of study
2018	Schwass DR, Lyons KM, Love R, Tompkins GR, Meledandri CJ.	Antimicrobial Activity of a Colloidal AgNP Suspension Demonstrated In Vitro against Monoculture Biofilms: Toward a Novel Tooth Disinfectant for Treating Dental Caries	Adv Dent Res	Serch noise
2017	Schwendicke F, Goestemeyer G.	Cost-effectiveness of root caries preventive treatments	J Dent	Different outcome

2014	Schwendicke, F., Meyer-Lueckel, H., Schulz, M., Doerfer, C.E., Paris, S.	Radiopaque tagging masks caries lesions following incomplete excavation in vitro	Journal of Dental Research	Different type of study
2020	Scully A.C., Yepes J.F., Tang Q., Downey T., Maupome G.	Utilization of Silver Diamine Fluoride by Dentists in the United States: A Dental Claims Review	Pediatric dentistry	Different type of study
2018	Seifo N, Al-Yaseen W, Innes N.	The efficacy of silver diamine fluoride in arresting caries in children	Evid Based Dent	Different type of study
2021	Seifo N, Cassie H, Radford JR, Innes NPT.	"I guess it looks worse to me, it doesn't look like there's been a problem solved but obviously there is": a qualitative exploration of children's and their parents' views of silver diamine fluoride for the management of carious lesions in children	BMC Oral Health	Different type of study
2019	Seifo N, Cassie H, Radford JR, Innes NPT.	Silver diamine fluoride for managing carious lesions: an umbrella review	BMC Oral Health	Different type of study
2020	Seifo N., Cassie H., Radford J., Innes N.	"It's really no more difficult than putting on fluoride varnish": a qualitative exploration of dental professionals' views of silver diamine fluoride for the management of carious lesions in children	BMC oral health	Different type of study
2020	Seifo N., Robertson M., MacLean J., Blain K., Grosse S., Milne R., Seeballuck C., Innes N.	The use of silver diamine fluoride (SDF) in dental practice	British dental journal	Different type of study
2011	Sequeira-Byron P, Lussi A.	Prevention of root caries	Evid Based Dent	Serch noise
2020	Seto J., Horst J.A., Parkinson D.Y., Frachella J.C., DeRisi J.L.	Enhanced Tooth Structure Via Silver Microwires Following Treatment with 38 Percent Silver Diamine Fluoride	Pediatric dentistry	Different type of study
2015	Sharma G, Puranik MP, K R S.	Approaches to Arresting Dental Caries: An Update	J Clin Diagn Res	Different type of study
1994	Shellis RP.	Effects of a supersaturated pulpal fluid on the formation of caries-like lesions on the roots of human teeth	Caries Res	Different outcome

2012	Shibata S, Suge T, Kimura T, Ishikawa K, Matsuo T.	Antibacterial activity of ammonium hexafluorosilicate solution with antimicrobial agents for the prevention of dentin caries	Am J Dent	Serch noise
2020	Sihra R., Schroth R.J., Bertone M., Martin H., Patterson B., Mittermuller B.-A., Lee V., Moffatt M.E., Klus B., Fontana M., Robertson L.	The Effectiveness of Silver Diamine Fluoride and Fluoride Varnish in Arresting Caries in Young Children and Associated Oral Health-Related Quality of Life	Journal (Canadian Dental Association)	Follow up < 12 mo.
2019	Silva, A.V.C., Teixeira, J.A., de Melo JÃºnior, P.C., de Souza Lima, M.G., de Oliveira Mota, C.C.B., Lins, E.C.C.C., Pereira, J.R.D., Gomes, A.S.L., Targino, A.G.R., Rosenblatt, A.	Remineralizing potential of nano-silver-fluoride for tooth enamel: An optical coherence tomography analysis	Pesquisa Brasileira em Odontopediatria e Clinica Integrada	Serch noise
2011	Sinha N, Gupta A, Logani A, Shah N.	Remineralizing efficacy of silver diamine fluoride and glass ionomer type VII for their proposed use as indirect pulp capping materials - Part II (A clinical study)	J Conserv Dent	Different type of sample
2018	Slayton R.L., Urquhart O., Araujo M.W.B., Fontana M., GuzmÃ¡n-Armstrong S., Nascimento M.M., NovÃ½ B.B., Tinanoff N., Weyant R.J., Wolff M.S., Young D.A., Zero D.T., Tampi M.P., Pilcher L., Banfield L., Carrasco-Labra A.	Evidence-based clinical practice guideline on nonrestorative treatments for carious lesions: A report from the American Dental Association	Journal of the American Dental Association (1939)	Different type of study
1975	Snawder, K.D., Gonzalez Jr., W.E.	Management of severely diseased primary anterior teeth.	ASDC journal of dentistry for children	Serch noise
2020	Soares-Yoshikawa, A.L., Cury, J.A., Tabchoury, C.P.M.	Fluoride concentration in SDF commercial products and their bioavailability with demineralized dentine	Brazilian Dental Journal	Different outcome
1963	Sognnaes, R.F.	The present status of caries research	The Journal of Prosthetic Dentistry	Different type of study
2021	Sorkhdini P, Crystal YO, Tang Q, Lippert F.	In vitro rehardening and staining effects of silver diamine fluoride with and without mucin on early enamel caries lesions	Am J Dent	Different type of study
2021	Sorkhdini P, Crystal YO, Tang Q, Lippert F.	The effect of silver diamine fluoride in preventing in vitro primary coronal caries under pH-cycling conditions	Arch Oral Biol	Different type of study

2020	Sorkhdini P, Gregory RL, Crystal YO, Tang Q, Lippert F.	Effectiveness of in vitro primary coronal caries prevention with silver diamine fluoride - Chemical vs biofilm models	J Dent	Different type of study
2016	Soxman J.A.	Noninvasive treatment for cavitated lesions in primary molars	General dentistry	Abstract or full-text not avable
2020	Splieth C.H., Banerjee A., Bottenberg P., Breschi L., Campus G., Ekstrand K.R., Giacaman R.A., Haak R., Hannig M., Hickel R., Juric H., Lussi A., Machiulskiene V., Manton D.J., Jablonski-Momeni A., Opdam N.J.M., Paris S., SantamarÃa R.M., Schwendicke F., Tassery H., Ferreira Zandona A., Zero D.T., Zimmer S., DomÃ©jean S.	How to Intervene in the Caries Process in Children: A Joint ORCA and EFCD Expert Delphi Consensus Statement	Caries research	Different type of study
2011	Splieth, C.H., Berndt, C., Alkilzy, M., Treuner, A.	Efficacy of semiannual topical fluoride application in schoolchildren	Quintessence International	Serch noise
2021	Srisomboon S, Kettratad M, Pakawanit P, Rojviriya C, Phantumvanit P, Panpisut P.	Effects of Different Application Times of Silver Diamine Fluoride on Mineral Precipitation in Demineralized Dentin	Dent J (Basel)	Different outcome
2009	Stanislawczuk, R., Amaral, R.C., Zander-Grande, C., Gagler, D., Reis, A., Loguercio, A.D.	Chlorhexidine-containing acid conditioner preserves the longevity of resin-dentin bonds	Operative Dentistry	Serch noise
2011	Stanislawczuk, R., Reis, A., Loguercio, A.D.	A 2-year in vitro evaluation of a chlorhexidine-containing acid on the durability of resin-dentin interfaces	Journal of Dentistry	Serch noise
2018	Sueyama, Y., Kaneko, T., Ito, T., Okiji, T.	Effect of lipopolysaccharide stimulation on stem cell-associated marker-expressing cells	International Endodontic Journal	Serch noise
2008	Suge, T., Kawasaki, A., Ishikawa, K., Matsuo, T., Ebisu, S.	Ammonium hexafluorosilicate elicits calcium phosphate precipitation and shows continuous dentin tubule occlusion	Dental Materials	Serch noise
2014	Sugiyama, M., Hattori, H., Inoue, T., Wakita, H., Hibi, H., Ueda, M.	Stem cells from human exfoliated deciduous teeth enhance recovery from focal cerebral ischemia in rats	Journal of Oral and Maxillofacial Surgery, Medicine, and Pathology	Serch noise

2021	Suguna, S., Jeevanandan, G., Rajeshkumar, S.	Cytotoxic effect of three different silver diamine fluoride: An in-vitro study	International Journal of Dentistry and Oral Science	Different type of study
2021	Sulyanto RM, Kang M, Srirangapatanam S, Berger M, Candamo F, Wang Y, Dickson JR, Ng MW, Ho SP.	Biomineralization of Dental Tissues Treated with Silver Diamine Fluoride	J Dent Res	Different outcome
1989	Swift, E.J.	In vitro Caries-inhibitory Properties of a Silver Cermet	Journal of Dental Research	Serch noise
1979	SyryÅ„ska, M.	Current views on the use of silver amalgams with added fluoride in the prevention of secondary caries	Czasopismo stomatologiczne	Serch noise
2021	Takahashi M, Matin K, Matsui N, Shimizu M, Tsuda Y, Uchinuma S, Hiraishi N, Nikaido T, Tagami J.	Effects of silver diamine fluoride preparations on biofilm formation of Streptococcus mutans	Dent Mater J	Different outcome
1993	Takahashi, K., Emilson, C.G., Birkhed, D.	Fluoride release in vitro from various glass ionomer cements and resin composites after exposure to NaF solutions	Dental Materials	Serch noise
2010	Tan HP, Lo EC, Dyson JE, Luo Y, Corbet EF.	A randomized trial on root caries prevention in elders	J Dent Res	Serch noise
2014	Targino AG, Flores MA, dos Santos Junior VE, de Godoy BenÃ© Bezerra F, de Luna Freire H, Galembeck A, Rosenblatt A.	An innovative approach to treating dental decay in children. A new anti-caries agent	J Mater Sci Mater Med	Different outcome
2018	Tedesco T.K., Gimenez T., Floriano I., Montagner A.F., Camargo L.B., Calvo A.F.B., Morimoto S., Raggio D.P.	Scientific evidence for the management of dentin caries lesions in pediatric dentistry: A systematic review and network meta-analysis	PLoS ONE	Different type of study
2013	Tegginmani, V.S., Goel, B., Uppin, V., Horatti, P., Vijay Kumar, L.S., Nainani, A.	Comparison of antibacterial activity of glass-ionomer cement and amalgam in class two restorations by streptococcus mutans count analysis at fixed intervals: An in vivo study	Journal of Contemporary Dental Practice	Serch noise
2018	Teixeira, J.A., Costa E Silva, A.V., Dos Santos, V.E., De Melo, P.C., Arnaud, M., Lima, M.G., Flores, M.A.P., Stamford, T.C.M., Pereira, J.R.D., Targino, A.G.R., Galembeck, A., Rosenblatt, A.	Effects of a New Nano-Silver Fluoride-Containing Dentifrice on Demineralization of Enamel and Streptococcus mutans Adhesion and Acidogenicity	International Journal of Dentistry	Serch noise

2016	Thanatvarakorn, O., Islam, S., Nakashima, S., Sadr, A., Nikaido, T., Tagami, J.	Effects of zinc fluoride on inhibiting dentin demineralization and collagen degradation in vitro: A comparison of various topical fluoride agents	Dental Materials Journal	Different type of study
1978	Thibodeau, E.A., Handelman, S.L., Marquis, R.E.	Inhibition and Killing of Oral Bacteria by Silver Ions Generated with Low Intensity Direct Current	Journal of Dental Research	Serch noise
2020	Thomas M.L., Magher K., Mugayar L., DÃ¡vila M., Tomar S.L.	Silver Diamine Fluoride Helps Prevent Emergency Visits in Children with Early Childhood Caries	Pediatric dentistry	Different outcome
2021	Thomas, C.S., Sharma, D.S., Sheet, D., Mukhopadhyay, A., Sharma, S.	Cross-sectional visual comparison of remineralization efficacy of various agents on early smooth surface caries of primary teeth with swept source optical coherence tomography: Cross-sectional remineralization assessment with SS-OCT	Journal of Oral Biology and Craniofacial Research	Different type of study
2020	Timms L., Sumner O., Deery C., Rogers H.J.	Everyone else is using it, so why isn't the UK? Silver diamine fluoride for children and young people	Community dental health	Different type of study
2020	Timms, L., Deery, C., Stevens, C., Rodd, H.	COVID-2019 Time to use silver diamine fluoride for Caries Arrest in general dental practice?	Dental Update	Different type of study
1978	Toolson, L.B., Smith, D.E.	A 2-year longitudinal study of overdenture patients. Part I: Incidence and control of caries on overdenture abutments	The Journal of Prosthetic Dentistry	Serch noise
2021	Toopchi S, Bakhurji E, Loo CY, Hassan M.	Effect of Light Curing on Silver Diamine Fluoride in Primary Incisors: A Microscopic Ex Vivo Study	Pediatr Dent	Different type of study
2021	Torres PJ, Phan HT, Bojorquez AK, Garcia-Godoy F, Pinzon LM.	Minimally Invasive Techniques Used for Caries Management in Dentistry. A Review	J Clin Pediatr Dent	Different type of study
2001	Touyz, L.Z.G., Amsel, R.	Anticariogenic effects of black tea (Camellia sinensis) in caries prone-rats	Quintessence International	Different type of study
2016	Trask PA.	Silver Diamine Fluoride: It's About Time!	J Calif Dent Assoc	Different type of study

2009	Triches, T.C., Cordeiro, M.M.R., Souza, J.G.M.V., Saltori, E.K., FranÃ§a, B.H.S.	Parental acceptance of the use of diamine silver fluoride in children aged 0 to 3 years in the city of Cascavel, PR, Brazil	Pesquisa Brasileira em Odontopediatria e Clinica Integrada	Different outcome
2019	Trieu A., Mohamed A., Lynch E.	Silver diamine fluoride versus sodium fluoride for arresting dentine caries in children: a systematic review and meta-analysis	Scientific reports	Different type of study
2021	Tripathi, P., Mengi, R., Gajare, S.M., Nanda, S.S., Wani, S.A., Kochhar, A.S.	Evaluation of Remineralizing Capacity of P11-4, CPP-ACP, Silver Diamine Fluoride, and NovaMin: An In Vitro Study	Journal of Contemporary Dental Practice	Different type of study
2011	Tschoppe, P., Meyer-Lueckel, H.	Mineral distribution of artificial dentinal caries lesions after treatment with fluoride agents in combination with saliva substitutes	Archives of Oral Biology	Different type of study
2020	Turton B., Patel J., Hill R., Sieng C., Durward C.	Healthy Kids Cambodia - A novel approach to triage for dental care in a population with extreme caries experience	Community dentistry and oral epidemiology	Serch noise
2015	Twetman S, Dhar V.	Evidence of Effectiveness of Current Therapies to Prevent and Treat Early Childhood Caries	Pediatr Dent	Different type of study
2015	Twetman S.	The evidence base for professional and self-care prevention--caries, erosion and sensitivity	BMC Oral Health	Different type of study
2020	Uchil SR, Suprabha BS, Suman E, Shenoy R, Natarajan S, Rao A.	Effect of three silver diamine fluoride application protocols on the microtensile bond strength of resin-modified glass ionomer cement to carious dentin in primary teeth	J Indian Soc Pedod Prev Dent	Different outcome
2019	Urquhart O, Tampi MP, Pilcher L, Slayton RL, Araujo MWB, Fontana M, GuzmÃ¡n-Armstrong S, Nascimento MM, NovÃ½ BB, Tinanoff N, Weyant RJ, Wolff MS, Young DA, Zero DT, Brignardello-Petersen R, Banfield L, Parikh A, Joshi G, Carrasco-Labra A.	Nonrestorative Treatments for Caries: Systematic Review and Network Meta-analysis	J Dent Res	Different type of study
2015	van der Kaaij, N.C.W., van der Veen, M.H., van der Kaaij, M.A.E., ten Cate, J.M.	A prospective, randomized placebo-controlled clinical trial on the effects of a fluoride rinse on white spot lesion development and bleeding in orthodontic patients	European Journal of Oral Sciences	Different outcome

2018	van Strijp G, van Loveren C.	No Removal and Inactivation of Carious Tissue: Non-Restorative Cavity Control	Monogr Oral Sci	Different type of study
2020	Vargas JP, Uribe M, OrtuÃ±o D, Verdugo-Paiva F.	Silver diamine fluoride compared to atraumatic restorative technique for the treatment of caries in primary and mixed first phase dentition	Medwave	Different type of study
2012	Vasquez, E., Zegarra, G., Chirinos, E., Castillo, J.L., Taves, D.R., Watson, G.E., Dills, R., Mancl, L.L., Milgrom, P.	Short term serum pharmacokinetics of diammine silver fluoride after oral application	BMC Oral Health	Different outcome
2021	Vennela E, Sharada J, Hasanuddin S, Suhasini K, Hemachandrika I, Singh PT.	Comparison of staining potential of silver diamine fluoride versus silver diamine fluoride and potassium iodide under tooth-colored restorations: An in vitro study	J Indian Soc Pedod Prev Dent	Different type of study
2013	Vichi, A., Margvelashvili, M., Goracci, C., Papacchini, F., Ferrari, M.	Bonding and sealing ability of a new self-adhering flowable composite resin in class I restorations	Clinical Oral Investigations	Serch noise
2020	Vinod D, Gopalakrishnan A, Subramani SM, Balachandran M, Manoharan V, Joy A.	A Comparative Evaluation of Remineralizing Potential of Three Commercially Available Remineralizing Agents: An In Vitro Study	Int J Clin Pediatr Dent	Different type of study
2018	Vinson LA, Gilbert PR, Sanders BJ, Moser E, Gregory RL.	Silver Diamine Fluoride and Potassium Iodide Disruption of In Vitro Streptococcus mutans Biofilm	J Dent Child (Chic)	Different type of study
2019	Vollu, A.L., Moreira, J.P.L., Luiz, R.R., Barja-Fidalgo, F., Fonseca-GonÃ§alves, A.	Survey of knowledge, attitudes and practices of brazilian dentists regarding silver diamine fluoride	Pesquisa Brasileira em Odontopediatria e Clinica Integrada	Different outcome
2021	Wakhloo T, Reddy SG, Sharma SK, Chug A, Dixit A, Thakur K.	Silver Diamine Fluoride Versus Atraumatic Restorative Treatment in Pediatric Dental Caries Management: A Systematic Review and Meta-analysis	J Int Soc Prev Community Dent	Different type of study
2017	Walsh, L.J.	Minimal intervention management of the older patient	British Dental Journal	Serch noise
1998	Wandern, A.	In vitro enamel effects of a resin-modified glass ionomer: Fluoride uptake and resistance to demineralization	Pediatric Dentistry	Serch noise

1991	Wang, N.-H., von der Lehr, W.N.	The direct and indirect techniques of making magnetically retained overdentures	The Journal of Prosthetic Dentistry	Serch noise
2015	Wang, X., Wang, B., Wang, Y.	Antibacterial orthodontic cement to combat biofilm and white spot lesions	American Journal of Orthodontics and Dentofacial Orthopedics	Serch noise
2020	Warren J.J., Thrap S., Starr D.	Dental caries treatment completed under general anesthesia among American Indian children in a northern plains tribal community	Journal of public health dentistry	Serch noise
2014	Wegehaupt, F.J., Buchalla, W., Sener, B., Attin, T., Schmidlin, P.R.	Cerium chloride reduces enamel lesion initiation and progression in vitro	Caries Research	Serch noise
2019	Weintraub J.A., Birken S.A., Burgette J.M., Lewis T.A., White B.A.	Use of the consolidated framework for implementation research to assess determinants of silver diamine fluoride implementation in safety net dental clinics	Journal of public health dentistry	Different type of study
2015	Wierichs RJ, Meyer-Lueckel H.	Systematic review on noninvasive treatment of root caries lesions	J Dent Res	Different type of study
2018	Wierichs RJ, Stausberg S, Lausch J, Meyer-Lueckel H, Esteves-Oliveira M.	Caries-Preventive Effect of NaF, NaF plus TCP, NaF plus CPP-ACP, and SDF Varnishes on Sound Dentin and Artificial Dentin Caries in vitro	Caries Res	Different type of study
2017	Wong A, Subar PE, Young DA.	Dental Caries: An Update on Dental Trends and Therapy	Adv Pediatr	Different type of study
2011	Wong MC, Lam KF, Lo EC.	Analysis of multilevel grouped survival data with time-varying regression coefficients	Stat Med	Serch noise
2005	Wong MC, Lam KF, Lo EC.	Bayesian analysis of clustered interval-censored data	J Dent Res	Serch noise
1993	Wood, R.E., Maxymiw, W.G., McComb, D.	A clinical comparison of glass ionomer (polyalkenoate) and silver amalgam restorations in the treatment of Class 5 caries in xerostomic head and neck cancer patients.	Operative dentistry	Serch noise

2017	Wright J.T., White A.	Silver Diamine Fluoride: Changing the Caries Management Paradigm and Potential Societal Impact	North Carolina medical journal	Different type of study
2016	Wu DI, Velamakanni S, Denisson J, Yaman P, Boynton JR, Papagerakis P.	Effect of Silver Diamine Fluoride (SDF) Application on Microtensile Bonding Strength of Dentin in Primary Teeth	Pediatr Dent	Different outcome
2020	Yalmaz, N., Ocak, M., Ã–kte, Z.	REMINERALIZATION OF PRIMARY MOLAR DENTINE WITH SILVER DIAMINE FLUORIDE AND SODIUM FLUORIDE: AN IN VITRO STUDY	Cumhuriyet Dental Journal	Different type of study
2019	Yamada Y, Nakamura-Yamada S, Umemura-Kubota E, Baba S.	Diagnostic Cytokines and Comparative Analysis Secreted from Exfoliated Deciduous Teeth, Dental Pulp, and Bone Marrow Derived Mesenchymal Stem Cells for Functional Cell-Based Therapy	Int J Mol Sci	Serch noise
1972	Yamaga R, Nishino M, Yoshida S, Yokomizo I.	Diammine silver fluoride and its clinical application	J Osaka Univ Dent Sch	Abstract or full-text not avable
2019	Yarmarkovich P , Alrayyes S , Lee HH , LeHew C , da Fonseca MA	SDF's Role in Reducing Urgent-Care Encounters While Awaiting General Anesthesia	Pediatr Dent	Different type of study
2021	Yawary R, Hegde S.	Silver Diamine Fluoride Protocol for Reducing Preventable Dental Hospitalisations in Victorian Children	Int Dent J	Different type of study
2014	Yetkiner, E., Wegehaupt, F., Wiegand, A., Attin, R., Attin, T.	Colour improvement and stability of white spot lesions following infiltration, micro-abrasion, or fluoride treatments in vitro	European Journal of Orthodontics	Different type of study
2020	Yin IX, Zhao IS, Mei ML, Li Q, Yu OY, Chu CH.	Use of Silver Nanomaterials for Caries Prevention: A Concise Review	Int J Nanomedicine	Different type of study
2020	Yin, I.X., Yu, O.Y., Zhao, I.S., Mei, M.L., Li, Q.L., Tang, J., Lo, E.C.M., Chu, C.H.	Inhibition of dentine caries using fluoride solution with silver nanoparticles: An in vitro study	Journal of Dentistry	Different type of study
2018	Young DA, Frostad-Thomas A, Gold J, Wong A.	Secondary Sjogren syndrome: A case report using silver diamine fluoride and glass ionomer cement	J Am Dent Assoc	Different outcome

2021	Young DA, Quock RL, Horst J, Kaur R, MacLean JK, Frachella JC, Duffin S, Semprum-Clavier A, Ferreira Zandona AG.	Clinical Instructions for Using Silver Diamine Fluoride (SDF) in Dental Caries Management	Compend Contin Educ Dent	Different outcome
2018	Yu OY, Mei ML, Zhao IS, Li QL, Lo EC, Chu CH.	Remineralisation of enamel with silver diamine fluoride and sodium fluoride	Dent Mater	Different type of study
2018	Yu OY, Zhao IS, Mei ML, Lo ECM, Chu CH.	Caries-arresting effects of silver diamine fluoride and sodium fluoride on dentine caries lesions	J Dent	Different type of study
2019	Zander V, Chan D, Sadr A.	Microcomputed Tomography Evaluation of Root Dentin Caries Prevention by Topical Fluorides and Potassium Iodide	Sensors (Basel)	Serch noise
1945	Zander, H.A., Smith, H.W.	Penetration of silver nitrate into dentin II	Journal of Dental Research	Serch noise
2013	Zavareh, F.A., Samimi, P., Birang, R., Eskini, M., Bouraima, S.A.	Assessment of microleakage of class V composite resin restoration following erbium-doped yttrium aluminum garnet (Er: YAG) laser conditioning and acid etching with two different bonding systems	Journal of Lasers in Medical Sciences	Serch noise
2009	Zehetbauer, S., Wojahn, T., Hiller, K.-A., Schmalz, G., Ruhl, S.	Resemblance of salivary protein profiles between children with early childhood caries and caries-free controls	European Journal of Oral Sciences	Serch noise
1996	Zhang C, Kimura Y, Matsumoto K.	The effects of pulsed Nd:YAG laser irradiation with fluoride on root surface	J Clin Laser Med Surg	Serch noise
2020	Zhang J, Sardana D, Li KY, Leung KCM, Lo ECM.	Topical Fluoride to Prevent Root Caries: Systematic Review with Network Meta-analysis	J Dent Res	Sdifferent type of study
2013	Zhang W, McGrath C, Lo EC, Li JY.	Silver diamine fluoride and education to prevent and arrest root caries among community-dwelling elders	Caries Res	Different outcome
2018	Zhao IS, Gao SS, Hiraishi N, Burrow MF, Duangthip D, Mei ML, Lo EC, Chu CH.	Mechanisms of silver diamine fluoride on arresting caries: a literature review	Int Dent J	Different type of study

2017	Zhao IS, Mei ML, Burrow MF, Lo EC, Chu CH.	Effect of Silver Diamine Fluoride and Potassium Iodide Treatment on Secondary Caries Prevention and Tooth Discolouration in Cervical Glass Ionomer Cement Restoration	Int J Mol Sci	Different outcome
2017	Zhao IS, Mei ML, Burrow MF, Lo EC, Chu CH.	Prevention of secondary caries using silver diamine fluoride treatment and casein phosphopeptide-amorphous calcium phosphate modified glass-ionomer cement	J Dent	Different type of study
2017	Zhao IS, Mei ML, Li QL, Lo ECM, Chu CH.	Arresting simulated dentine caries with adjunctive application of silver nitrate solution and sodium fluoride varnish: an in vitro study	Int Dent J	Serch noise
2021	Zhao IS, Xue VW, Yin IX, Niu JY, Lo ECM, Chu CH.	Use of a novel 9.3-um carbon dioxide laser and silver diamine fluoride: Prevention of enamel demineralisation and inhibition of cariogenic bacteria	Dent Mater	Serch noise
2020	Zhao IS, Yin IX, Mei ML, Lo ECM, Tang J, Li Q, So LY, Chu CH.	Remineralising Dentine Caries Using Sodium Fluoride with Silver Nanoparticles: An In Vitro Study	Int J Nanomedicine	Different type of study
2019	Zhao, I.S., Chu, S., Yu, O.Y., Mei, M.L., Chu, C.H., Lo, E.C.M.	Effect of silver diamine fluoride and potassium iodide on shear bond strength of glass ionomer cements to caries-affected dentine	International Dental Journal	Different outcome
2013	Zhi, Q.H., Lo, E.C.M., Kwok, A.C.Y.	An in vitro study of silver and fluoride ions on remineralization of demineralized enamel and dentine	Australian Dental Journal	Different type of study
2014	Zingler, S., Pritsch, M., Wrede, D.J., Ludwig, B., Bister, D., Kneist, S., Lux, C.J.	A randomized clinical trial comparing the impact of different oral hygiene protocols and sealant applications on plaque, gingival, and caries index scores	European Journal of Orthodontics	Serch noise
2018		Policy on the Use of Silver Diamine Fluoride for Pediatric Dental Patients	Pediatric dentistry	Different type of study
2018		Use of Silver Diamine Fluoride for Dental Caries Management in Children and Adolescents, Including Those with Special Health Care Needs	Pediatric dentistry	Different type of study

Table S3. Studies studies excluded after full-text reading.

Publication Year	Author Names	Title	Source title	Reason for exclusion
2020	Al-Nerabieah, Z., Arrag, E.A., Comisi, J.C., Rajab, A.	Effectiveness of a novel nano-silver fluoride with green tea extract compared with silver diamine fluoride: A randomized, controlled, non-inferiority trial	International Journal of Dentistry and Oral Science	Not data for posterior teeth
2017	Anggraini, R., Darwita, R.R., Adiatman, M.	The effectiveness of silver diamine fluoride and propolis fluoride in arresting caries on primary teeth: A study on kindergarten students in west jakarta, indonesia	Journal of International Dental and Medical Research	Not data for posterior teeth
2019	Bernstein R.S., Johnston B., Mackay K., Sanders J.	Implementation of a primary care physician-led Cavity Clinic using silver diamine fluoride	Journal of public health dentistry	Not data for posterior teeth
2021	Chaurasiya A., Gojanur S.	Evaluation of the clinical efficacy of 38% silver diamine fluoride in arresting dental caries in primary teeth and its parental acceptance	Journal of the Indian Society of Pedodontics and Preventive Dentistry	Not data for posterior teeth
2018	Clemens J., Gold J., Chaffin J.	Effect and acceptance of silver diamine fluoride treatment on dental caries in primary teeth	Journal of public health dentistry	Not data for posterior teeth
2012	Dos Santos Jr. V.E., De Vasconcelos F.M.N., Ribeiro A.G., Rosenblatt A.	Paradigm shift in the effective treatment of caries in schoolchildren at risk	International Dental Journal	Not data for posterior teeth
2016	Duangthip D., Chu C.H., Lo E.C.	A randomized clinical trial on arresting dentine caries in preschool children by topical fluorides--18 month results	Journal of Dentistry	Not data for posterior teeth
2018	Duangthip D., Wong M.C.M., Chu C.H., Lo E.C.M.	Caries arrest by topical fluorides in preschool children: 30-month results	Journal of Dentistry	Not data for posterior teeth
2019	Fahmi M.Q.M., Abbas M.J., Almallah L.A.A.	Topical effect of silver diamine fluoride in arresting dental caries in primary teeth	Indian Journal of Public Health Research and Development	Not data for posterior teeth

2016	Fung MHT, Duangthip D, Wong MCM, Lo ECM, Chu CH.	Arresting Dentine Caries with Different Concentration and Periodicity of Silver Diamine Fluoride	JDR Clin Trans Res	Same sample included in another article with a longer follow-up
2020	Gao S.S., Chen K.J., Duangthip D., Wong M.C.M., Lo E.C.M., Chu C.H.	Preventing early childhood caries with silver diamine fluoride: Study protocol for a randomised clinical trial	Trials	Not data for posterior teeth
2019	Gao S.S., Duangthip D., Wong M.C.M., Lo E.C.M., Chu C.H.	Randomized Trial of Silver Nitrate with Sodium Fluoride for Caries Arrest	Journal of Dental Research	Same sample included in another article with a longer follow-up
1994	McDonald SP, Sheiham A.	A clinical comparison of non-traumatic methods of treating dental caries	Int Dent J	Full text unavailable
2018	Milgrom P., Horst J.A., Ludwig S., Rothen M., Chaffee B.W., Lyalina S., Pollard K.S., DeRisi J.L., Mancl L.	Topical silver diamine fluoride for dental caries arrest in preschool children: A randomized controlled trial and microbiological analysis of caries associated microbes and resistance gene expression	Journal of dentistry	Not data for posterior teeth
2021	Rehim Y.M.A., Waly N.G., Abdelgawad F., Elmasry E.S.	Pain, new caries and failure of carious primary teeth after application of silver diamine fluoride versus sodium fluoride varnish: A randomized clinical trial	Indian Journal of Public Health Research and Development	Not data for posterior teeth
2020	Turton B, Horn R, Durward C.	Caries arrest and lesion appearance using two different silver fluoride therapies with and without potassium iodide: 6-month results	Heliyon	Same sample included in another article with a longer follow-up
2021	Turton B., Horn R., Durward C.	Caries arrest and lesion appearance using two different silver fluoride therapies on primary teeth with and without potassium iodide: 12-month results	Clinical and experimental dental research	Not data for posterior teeth
2009	Yee R., Holmgren C., Mulder J., Lama D., Walker D., Helderman W.V.P.	Efficacy of silver diamine fluoride for arresting caries treatment	Journal of Dental Research	Not data for posterior teeth
2021	Raskin S.E., Tranby E.P., Ludwig S., Okunev I., Frantsve-Hawley J., Boynes S.	Survival of silver diamine fluoride among patients treated in community dental clinics: a naturalistic study	BMC oral health	Follow-up < 12 m.

Table S4. Summary statistics, results for each study (fixed-effect model)

Authors (Year)	N	n1	n2	ES (LL-UL)	Sig	R-Sig
Abdellatif et al. (2021)	123	49	74	0.85 (-0.37-2.07)	0.17	0.43
Mabangkhru et al. (2020)	646	279	367	0.38 (0.17-0.59)	0.00	0.91
Tirupathi et al. (2019)	147	76	71	-0.13 (-0.56-0.29)	0.53	0.02
Vollú et al. (2019)	111	63	48	0.10 (-0.80-1.01)	0.82	0.60
Fung et al. (2018)	1505	738	767	0.37 (0.27-0.47)	0.00	0.72

N = Total sample size; **n1** = Group A sample size; **n2** = Group B sample size; **ES (LL-UL)** = Effect Size (95%CI Lower Limit- 95%CI Upper Limit); **Sig** = Statistical significance 2-tailed; **R-Sig**= Statistical significance for standardized residual
